# Improving performance of mammalian microRNA target prediction

**DOI:** 10.1186/1471-2105-11-476

**Published:** 2010-09-22

**Authors:** Hui Liu, Dong Yue, Yidong Chen, Shou-Jiang Gao, Yufei Huang

**Affiliations:** 1 SIEE, China University of Mining and Technology, Xuzhou, Jiangsu, China; 2 Department of ECE, University of Texas at San Antonio, USA; 3 Department of Pediatrics, University of Texas Health Science Center at San Antonio, USA; 4 Department of Epidemiology and Biostatistics, University of Texas Health Science Center at San Antonio, USA; 5 Greehey Children's Cancer Research Institute, University of Texas Health Science Center at San Antonio, USA

## Abstract

**Background:**

MicroRNAs (miRNAs) are single-stranded non-coding RNAs known to regulate a wide range of cellular processes by silencing the gene expression at the protein and/or mRNA levels. Computational prediction of miRNA targets is essential for elucidating the detailed functions of miRNA. However, the prediction specificity and sensitivity of the existing algorithms are still poor to generate meaningful, workable hypotheses for subsequent experimental testing. Constructing a richer and more reliable training data set and developing an algorithm that properly exploits this data set would be the key to improve the performance current prediction algorithms.

**Results:**

A comprehensive training data set is constructed for mammalian miRNAs with its positive targets obtained from the most up-to-date miRNA target depository called miRecords and its negative targets derived from 20 microarray data. A new algorithm SVMicrO is developed, which assumes a 2-stage structure including a site support vector machine (SVM) followed by a UTR-SVM. SVMicrO makes prediction based on 21 optimal site features and 18 optimal UTR features, selected by training from a comprehensive collection of 113 site and 30 UTR features. Comprehensive evaluation of SVMicrO performance has been carried out on the training data, proteomics data, and immunoprecipitation (IP) pull-down data. Comparisons with some popular algorithms demonstrate consistent improvements in prediction specificity, sensitivity and precision in all tested cases. All the related materials including source code and genome-wide prediction of human targets are available at http://compgenomics.utsa.edu/svmicro.html.

**Conclusions:**

A 2-stage SVM based new miRNA target prediction algorithm called SVMicrO is developed. SVMicrO is shown to be able to achieve robust performance. It holds the promise to achieve continuing improvement whenever better training data that contain additional verified or high confidence positive targets and properly selected negative targets are available.

## Background

MicroRNAs (miRNAs) are single-stranded non-coding RNAs with about 22 nucleotides in length[1] known to mainly inhibit target translation or cleave target mRNA by binding to the complementary sites in the 3' untranslated region (UTR) of targets. miRNAs have been shown and are speculated to play many important post-transcriptional regulatory roles in a wide range of biological processes and diseases including development, stress responses, viral infection, and cancer[2]. Despite rapid advance in miRNA research, the detailed functions and regulatory mechanisms of most of miRNAs are still poorly understood. To gain better understanding, an important task herein is to identify miRNAs' regulatory targets. However, the current knowledge about the known targets is disproportional to that of the known miRNAs. In miRBase, 969 human miRNAs are annotated; in contrast, only 815 targets of 121 human miRNAs are recorded in the most up-to-date target database miRecords[3]. Given that targets of each miRNA could be hundreds, the reported number of verified targets accounts for only a very small fraction of the potential targets. This fact greatly underscores the urgent need to develop effective target identification methods for genome-wide target discovery.

Considerable advances have been made in computational target prediction[4] and many algorithms have been proposed including TargetScan[5], PicTar[6], miRanda[7], PITA[8], DIANA-microT[9], RNAhybrid[10], microInspector[11], MovingTargets[12], rna22[13], NBmiRTar[14] and Nucleus[15]. These algorithms make predictions mainly based on various important features of miRNA-target nucleotide sequence interaction. Although different algorithms utilize different sets of features, a few important features including "seed region complementary", "binding free energy", and "sequence conservation" are among the most common ones. Using different features will result in different prediction performance and a central goal of various algorithms concerns the selection of most discriminative features that can lead to better prediction accuracy. A promising direction there is within the data driven framework, where the features are partially or entirely determined by training using the training data composed of validated positive and negative targets. Algorithms including MirTarget[16], miTarget[17], and TargetBoost[18] are data driven algorithms and developed based on training. Given sufficient training data, the data driven algorithms hold the promise to provide accurate prediction, since they have the ability to uncover important features from data that cannot be easily observed otherwise.

However, the existing algorithms have poor prediction specificity and sensitivity[19,20]. The performance deficiency is partially due to the poor understanding of the precise mechanisms underlying miRNA-target interaction [1,21], and therefore, the adopted features of the rules are not yet as specific and sensitive as needed. Besides, high quality training data essential for training data-driven algorithms is greatly lacking. For many algorithms, the positive training data are based on a very small number or even one of validated targets and thus hardly include important features relevant to different aspects of miRNA-target interactions; these problems hamper the ability of the data-driven algorithms to select discriminative features. Many others also select the positive targets from down-regulated genes in an mRNA microarray data of over-expressing a miRNA. However, since protein inhibition is considered as the primary function, any reduction at the mRNA level measured by microarray is likely due to the secondary effect of miRNA regulation. Consequently, these training data are neither specific since many under-expressed genes may not be targets, nor sensitive since many targets might not be under-expressed at the mRNA level. It is apparent that constructing a richer and more reliable training data set is the key to improve the performance of the current data-driven algorithms.

The aim of this paper is to improve the sensitivity and specificity of target prediction by constructing a comprehensive training dataset and developing a support vector machine (SVM)[22,23] algorithm that exploits extensive binding features. Particularly, we take advantage of the most updated miRNA target depository called miRecords to construct positive training data set. In addition, we derive the negative data set based on 20 microarray data, each generated by over-expressing a different miRNA. With this more diverse, higher quality, and larger quantity training data set, we develop a more sophisticate two-stage SVM based algorithm called SVMicrO. In SVMicrO, 113 and 30 features were extracted to survey the potential binding sites and the UTR characteristics, respectively. A feature selection step is introduced to select most discriminative features for site and UTR. Comparison with several popular target prediction algorithms are performed based on training data and results from high confidence experiments including IP pull down and proteomics experiments. All these investigations indicate that SVMicrO achieves consistently improved sensitivity, specificity, and precision, which proves it to be a competitive alternative to the existing sequenced-based algorithms.

## Methods

### The algorithm of SVMicrO

**Figure 1:**
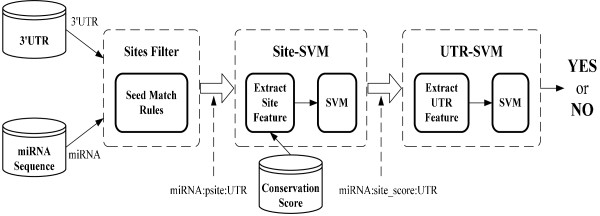
**The block diagram of SVMicrO**. SVMicrO includes three steps. First, a site filter is applied to find the potential binding sites of the probing miRNA. Second step, Site-SVM extracts features from each potential site and assigns a score to indicate the prediction confidence of the site as a true site. Final step, the site scores together with other UTR features are considered by the UTR-SVM to produce the final prediction of the UTR as a target.

The structure of SVMicrO is shown in Figure 1, which includes three steps. First, a site filter is applied, which uses the miRNA sequence to scan through the 3'UTR sequence for the potential binding sites of the probing miRNA. This filter is introduced to improve the efficiency of SVMicrO. The goal is to select the potential sites with high sensitivity since the sensitivity of the entire algorithm is upper-bounded by the sensitivity of the filter. In contrast, false positive sites identified by the seed match rules can be reduced by site-SVM and UTR-SVM. Many of the exiting algorithms also include a filter step, most of which rely on the presence of a 6-mer match in the seed region. However, by testing against the true binding sites and target pairs in the training data (see Construction of training data section for details), we find that more than 20% miRNA-site and 20% miRNA-target pairs do not possess the 6-mer seed match. This also implies that the existing target prediction algorithms relying on perfect seed match will result in a reduction of sensitivity regardless how good the later prediction is.

As a result, a looser seed match rule should be used to gain higher sensitivity. However, the seed match rules should be not too loose to introduce too many false positives; otherwise they would increase the computation burden of the subsequent SVMs. Out of these considerations, we examine the different combinations of nucleotides match statuses in seed region by considering TargetScan seed match rule [5], the results from [24] and our own investigation on the experimentally validated sites as well as targets in miRecords. Among different combinations, the following 5 seed match rules achieve near 96% sensitivity on both experiment validated targets and sites, while introducing less false positive sites than other combinations. Thus, regions of the 3'UTR sequence that obey one of the seed match rules are considered as potential sites.

(1) There are more than 4 continuous W-C matches, or

(2) There are more than 5 continuous matches (including G:U pair) and more than 2 continuous W-C matches, or

(3) There are more than 6 matches in total and 3 continuous W-C matches; no gap allowed, or

(4) 2~4 nucleotides of miRNA are W-C match, there is more than 3 W-C matches and more than 4 matches in total; no gap allowed, or

(5) There are more than 5 matches and 5 W-C matches, and only one gap is allowed on either miRNA sequence or 3'UTR.

Specifically, W-C match stands for Watson-Crick match, while match denotes Watson-Crick match or G:U wobble pair. The sensitivity of proposed filtering rules is evaluated by the training data and shown to be around 96% for both site and UTR (Additional file 1 Table 1), thus satisfying our goal of achieving higher sensitivity for the filter.

In the subsequent step, the potential sites identified by the filter are subjected to the Site-SVM, which extracts features from each site, and assigns a score to indicate the prediction confidence of the site as a true site. in the final step, the site scores together with other UTR features are considered by the UTR-SVM to produce the final prediction of the UTR as a target.

### Features extraction

Two different types of features representing important and possible binding characteristics were extracted for the Site- and UTR-SVM. A total of 113 site features and 30 UTR features are extracted.

#### Binding Structure definition

**Figure 2:**
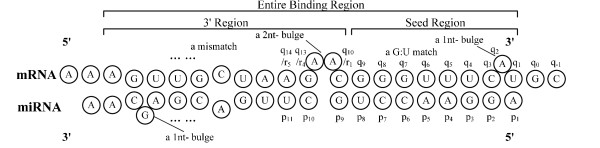
**Binding structure and regional definition of miRNA and target site**.

To define features, we first provide the mathematical definition of miRNA-site binding as in Figure 2. For a given miRNA sequence of length *M*, let *p *= {*p*_1_, ..., *p_m_*, ..., *p_M_*} denote its nucleotide composition, where *p_m _*∈ ***NT ***represents the nucleotide content at the *m*th position from its 5' end and ***NT ***= {*A*, *C*, *G*, *U*}. For a binding site of length *N*, let *q *= {*q*_1_, ..., *q_n_*, ..., *q_N_*} indicate its nucleotide composition from 3' end, where *q*_1 _is the nucleotide corresponding to *p*_1 _in miRNA. Naturally, {*q*_0_, ..., *q*_-1_, ...} stands for the 3' context of binding region.

Since based on the current consensus, the seed sequence of miRNA complements with UTR much better than the rest, we divide the entire binding site into two sub-regions, which are the seed binding region and the 3' binding region. From miRNA point of view, {*p*_1_, ..., *p*_8_} belong the seed binding region while {*p*_9_, ..., *p*_20_} belong to the 3' binding region. From mRNA's perspective, {*q*_1_, ..., *q*_*n*_} belong to the seed binding region and {*q*_*n*+1_, *q*_*n*+2_, ...} belong to the 3' binding region, respectively, where *q_n _*is the nucleotide of UTR that pairs with *p*_8 _of miRNA. We also use {*r*_1_, *r*_2_, ...} to denote {*q*_*n*+1_, *q*_*n*+2_, ...} in the following description. Moreover, only first 20 nucleotides of miRNA are consider in the feature extraction step in our algorithm, and thus the last nucleotides in {*r*_1_, *r*_2_, ...} pairs with *p*_20 _of miRNA.

#### Site Features

7 groups of site features are extracted to describe the characteristics of target recognition within a site (See Additional file 1 Table 2).

##### Perfect seed match features

Perfect seed match is widely used for binding site prediction in many target prediction algorithms. We survey 6 types of perfect seed matches (Table 1) and define the corresponding features as the existence of the respective match in the site.

**Table 1:** Definition of Perfect Seed Match

Seed Match Type	Description
6mer	If *p*_2 _~ *p*_7 _W-C complement.

7mer-A1	If *p*_2 _~ *p*_7 _W-C complement, *q*_1 _is A.

7mer-m1	If *p*_1 _~ *p*_7 _W-C complement.

7mer-m8	If *p*_2 _~ *p*_8 _W-C complement.

8mer-A1	If *p*_2 _~ *p*_8 _W-C complement, *q*_1 _is A.

8mer-m8	If *p*_1 _~ *p*_8 _W-C complement.

##### Pair-wise binding structure features

Past research shows that miRNA binding varies according to the position of the binding structure. In order to observe these characteristics, a modified version of RNAduplex [25] named miRNAbind is developed, which can be found in the SVMicrO package on the provided paper website. miRNAbind uses RNAduplex to generate the required secondary structure of miRNA binding similar to that in Figure 2 and it provides additional information including binding energy of seed region, binding energy of 3' region, exact boundaries of each regions, etc. Based on the secondary structure, four types of nucleotide matches are defined as W-C match, G-U match, mismatch, and gap. Subsequently, the match status of each nucleotide, represented by integer 1 to 4, as well as the content of each 2 mer, represented by integer 1 to 16, from *p*_1 _to *p*_20 _are extracted. There are totally 39 pair-wise binding structure features.

##### Regional binding structure features

To investigate the local binding characteristics, the sub-regions defined above and the total regions are used. For each region, the total numbers of W-C matches, G-U matches, mismatches, and gaps are counted as regional features. Additionally, to the reveal the bulge structure on mRNA, the numbers of bulged structures and bulged nucleotides in each binding region are also counted as 2 additional features. There are totally 18 features in this group.

##### Conservation features

To investigate the conservation characteristics of sites, the binding region is again divided into 3 sub-regions, which are the seed binding region, 5' context region ({*r*_1_, ⋯, *r*_10_}), and 3' context region ({*q*_0_, ⋯, *q*_-9_}). The conservation score of each nucleotide in the site is then obtained from the phastCons28way table (See Additional file 1 S.2) in the UCSC Gene Table. The conservation scores of each region are then defined as the average conservation scores of the respective regions.

##### Energy features

It is believed that miRNA-target binding forms a stable low energy hybrid. Hence, the more stable the binding structure is, the more likely the site is to be a true binding site. The binding energy features of the seed region, 3' region and total region are thus evaluated by miRNAbind. Moreover, the accessibility defined in PITA [8] is also adopted as another energy feature, which is introduced to evaluate the openness attribute of the secondary structure of a potential site.

##### Seed context features

Context region stands for the contiguous upstream and downstream sequences of the seed region. It has been reported that seed region preferentially resides within a locally AU-rich context [5]. To this end, two 10-nt long sequences, {*r*_1_, ⋯, *r*_10_} and {*q*_0_, ⋯, *q*_-8_}, on both ends of seed binding regions are isolated as context regions. The single nucleotide and 2-mer compositions are then recorded for each context regions to obtain 20 context features. Moreover, the nucleotide compositions of all positions in these 2 regions are regarded as another 20 context nucleotide type features.

##### Site location features

It is also reported that binding sites are more frequently observed at the two ends of a 3'UTR but not too close to the stop codon [5]. To reflect this point, we define 3 features including the distance from the potential site to stop codon, the distance from the potential site to the nearest end of 3'UTR, and the ratio of the distance from the potential site to the nearest end over the length of 3'UTR.

#### UTR Features

3 groups of UTR features are extracted to describe the characteristics of target recognition within the 3'UTR (See Additional file 1 Table 3).

##### Length of 3'UTR

Since a target 3'UTR includes multiple binding sites, the length of the 3' UTR presumably affects miRNA targeting. We investigate the length of 3'UTR in our training data set and the result shows that the positive targets on average have longer length than the negative targets. So we define the length of 3' UTR as a UTR feature.

##### Site density features

It has been demonstrated that the effectiveness of binding reduces if the distances among the sites are large [5,24]. To reflect this fact, global site density feature is calculated as the ratio of the number of potential/positive binding sites over the length of 3'UTR. Also, a 100-nt long window is used to identify a region with the maximum number of potential/positive binding sites and these maximum numbers are recorded as 2 features.

##### Binding site score features

The score produced by the Site-SVM for each candidate site can be regarded as the prediction confidence for this site. Consequently, the higher the confidence of site predictions, the more likely the UTR is to be a target. Again, the potential sites are the sites identified by the filter, and the positive sites are the potential sites predicted positive by the Site-SVM (SVM score >0). The total score of positive sites, the number of potential sites, the number of positive sites, etc as defines in Additional file 1 Table 4 are defined as 25 features.

### Construction of training data

Data used for training and testing should include both positive and negative miRNA-site and miRNA-target pairs for Site- and UTR-SVM. RefSeq information for human, mouse and rat have been downloaded from UCSC Genome Browser mySQL database. The sequences of 3'UTRs and the conservation scores of each nucleotide in the 3'UTR have been retrieved from the UCSC Genome Browser either. A local database for the sequences of 3'UTRs and the conservation scores have been built. A local miRNA sequences database has also been created based on miRBase V12.0. The positive and negative data sets are obtained as follows:

#### Positive data

The positive data are obtained from miRecords, which records most up-to-data experimentally verified targets. Since our goal is to predict targets of mammalian miRNAs, we only focus on the records of human (1020 records), mouse (166 records), and rat (133 records). To ensure the fidelity of training data, all the miRNA sequences are mapped to miRBase and all binding site sequences are aligned to the corresponding 3'UTRs; site records with irresolvable problems are removed. For some miRNA-target pairs that share the same miRNA and 3'UTR region, only one record is retained. Finally, 324 miRNA-site pairs are obtained from 187 miRNA-target pairs, and 709 additional miRNA-target pairs are also extracted but without site information.

#### Negative data

Currently, the negative data are almost nonexistent in any annotated database. In this case, miRNA over-expression microarray data are consulted and we assume that negative targets are less likely to be under-expressed under miRNA over-expression. To provide diversity of the negative data, we collected 20 miRNA over-expression microarray data from NCBI Gene Expression Omnibus (Additional file 1 Table 4). To generate the high quality negative data, we only consider the most confident up-regulated genes by restricting the differential expression *p *value, the fold change, and consistency of the samples over time whenever available. After the negative miRNA-target pairs are derived, the negative miRNA-site pair data are generated by the site filter. In the end, we obtain 3542 negative miRNA-target pairs. (See Additional file 1 S.5 for detailed discussion)

### Feature selection and training

For both Site- and UTR-SVM, RBF is chosen as the kernel function. 5-fold cross validation is carried out to train the parameters and select features for both SVMs. Due to the imbalance between the positive and negative data, the cost ratio factor is introduced in the SVMs. To measure the prediction accuracy, F score is adopted, which is a unified measurement of the prediction precision *p *and sensitive *r*, i.e.

$$F_{\beta }=(1+\beta^{2})\frac{p\cdot r}{\beta^{2}\cdot p+r}$$

where *p *and *r *represent the prediction precision and sensitivity, respectively, and *β *≥ 0 is a pre-specified weight that defines the relative importance between precision and sensitivity. In this case, *β *= 1, which puts equal importance on precision and sensitivity in defining the final prediction accuracy.

In each round of cross validation, a sequential forward search algorithm is implemented for feature selection based on the ranked features by minimal redundancy maximal relevance (mRMR) algorithm [26]. In a nutshell, the mRMR algorithm is designed to choose a subset of features that have the highest relevance to the target class while maintaining the minimal redundancy. Particularly, the redundancy measures the correlation among features. Given a feature set *S *with *m *features {*x_i_*}, *i*=1, ..., *m*, the relevance D with the target class *c *is defined by

(1) $$D=\frac{1}{\left|S\right|}\sum_{x_{i}\in S}I(x_{i};c)$$

and the redundancy *R *among features in *S *is given by

(2) $$R=\frac{1}{|S{|}^{2}}\sum_{x_{i},x_{j}\in S}I(x_{i};x_{j})$$

where *I*(·,·) is the mutual information and defined by

(3) $$I(x;y)=\iint p(x,y)\log\frac{p(x,y)}{p(x)p(y)}dxdy$$

where *p*(*x*), *p*(*y*) and *p*(*x*, *y*) denote the marginal and jointly probability density functions, respectively. mRMR selects the minimal redundant maximal relevant feature set that maximizes the objective Φ(*D*, *R*) = D-R. The optimization can be achieved by a greedy search that iterates among features individually. A side product to the final optimal set is a feature rank list. Based on this rank list, a sequential forward search algorithm is applied to site- and UTR-SVM through cross validation on the training data to determine the 21 optimal site features (Table 2) and 18 optimal UTR features (3). Based on these optimal features, SVMicrO can achieve the best prediction accuracy in terms of F score.

**Table 2:** The optimal site feature set

	Feature		Feature
1	6mer seed match	11	Number of matches in total region

2	Conservation score of 3' context region	12	Binding energy of seed region

3	Number of matches in seed region	13	Seed conservation score

4	7mer_A1 seed match	14	p7 match status

5	7mer_m8 seed match	15	Context nt type of r1

6	7mer_m1 seed match	16	Binding energy of total region

7	8mer_A1 seed match	17	conservation score of 5' context region

8	Accessibility energy	18	Number of mismatches in seed region

9	8mer_m1 seed match	19	p5 match status

10	6^th ^2mer status	20	p2 match status

		21	p12 match status

At the same time of feature selection, a 2-D grid search is carried out to optimize the parameters of SVM. Specifically, two parameters need to be optimized including the penalty constant *C *of SVM and the parameter γ of the RBF kernel. Refer to [27] for a detailed discussion regarding the definition of these parameters. The entire cross validation is implemented by C language based on SVMlight v6.01 http://svmlight.joachims.org/. At the end of cross validation, the optimized SVM and the feature sets are recorded.

## Results

### Investigation of site features

The 21 optimal site features are resulted from the sequential forward search feature selection applied to the site training data (Table 2). Close examination of these features lead the following observations.

#### 7mer and 8mer seed matches are sufficient for miRNA site recognition

Overall, seed region is clearly the most important region as 13 out of the 21 optimal features are related to the seed region. Among the different categories of features, seed match features account for a large portion of the optimal features with 6-mer seed match ranked at the top. The histograms of various seed match features are plotted in Additional file 1 Figure 3. The most discriminative feature *6mer seed match *is present in around 80% true target sites but absent in 95% negative sites. All those seed type match features are also among the top ranked. These results echo the general belief that the seed matches are among the most important mechanisms for miRNA target recognition. However, the histograms of 7mer and 8mer matches are much less distinct between positive and negative data than those of the 6mer. Interestingly, almost no negative target sites possess these seed type match features; this implies that using these features on top of *6mer seed match *reduces the false positive rate, although they are not as nearly sensitive as *6mer seed match*. This fact is also demonstrated by the ROC curve of site-SVM in Additional file 1 Figure 10. From the perspective of miRNA site recognition mechanism, these data suggest that the 7mer and 8mer matches are unique to miRNA site recognition; however, miRNA does not always employ these mechanisms in target recognition.

#### Conservation of 3'context region downstream of seed is of higher importance

As expected, all conservation features including those of the seed, 5' and 3' context regions are important features. Unexpectedly, the conservation of the 3' context region, or 10 nts downstream of the seed region, ranks the 2nd and plays more important roles than the seed conservation, which ranks only the 13th in the list. Many existing algorithms including TargetScan rely on seed conservation but none of them consider the conservation of 3' context region. This finding points to the importance of downstream and upstream regions of the binding sites. Sequence motifs of Argonaute protein, ALG-1, binding have been revealed by cross-linking IP to preside in these regions. We hypothesize that the significance of the conservation features in the context regions is a result of Argonaute binding to UTR.

#### Accessibility energy and seed binding energy are important features

Energy features include accessibility energy and binding energies of the context region, seed, and total region. Among them, only accessibility energy and binding energy of seed regions are determined to be among the optimal features. It is not surprising to see the seed binding energy in this list, which recapitulates the importance of the seed region. However, accessibility energy feature is determined to be more important (ranked the 8^th^); this fact stresses that the 2^nd ^structure of potential site can considerably influence the ability of miRNA binding. Currently, only PITA assesses the accessibility energy. This finding advocates the inclusion of this feature for target prediction.

### Predicted site characteristics of miR-1

**Figure 3:**
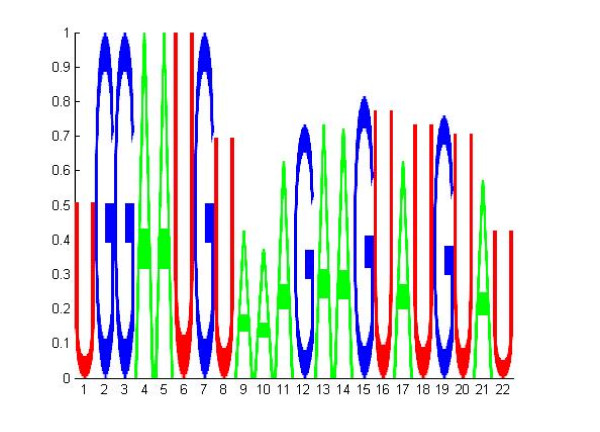
**Binding sequence logo of miR-1 predicted by Site-SVM**. The 22 nucleotides of miR-1 sequence are plotted from 5' to 3'. The height of each nucleotide is proportional to the probability of binding to the site.

To further demonstrate the ability of site-SVM to reveal insights about miRNA binding, we apply the site-SVM to predict the binding sites of miR-1 in 75 validated positive targets obtained in miRecords. Although they are reported positive targets, no binding sites are reported. This is a common scenario especially prevalent for high throughput screening of miRNA targets and the question often concerns the binding characteristics of a miRNA. For the 75 miR-1 targets, a total of 155 sites (or 2.07 sites/UTR) are predicted by site-SVM. A binding matrix is constructed based on the predicted sites with the *ij*-th element being 1 if the *j-*th nucleotide of miR-1 is paired to the *i*-th site, and 0, otherwise. Based on the binding matrix, the empirical probability of binding can be obtained for each of the nucleotides in miR-1 sequence and a binding sequence logo is generated by the TarLogo program in the SVMicrO suite and plotted in Figure 3 to depict these binding probabilities. Figure 3 reveals two regions in miR-1 sequences that are likely to be responsible for binding to its target. The first region corresponds to the 6-mer seed from nt 2-6, and 100% probabilities in this region suggests miR-1 has perfect 6-mer pairing with every sites. The second region stretches from nt 12-20. The binding probabilities are not as high as those in the seed region but there is still a relative high chance of binding compared with the rest of the sequence. A close look into the secondary structure of binding at each predicted sites reveals that there is an average of 7 bulges and mismatches in this region of the site; the largest number of bulges and mismatches is 25 and the smallest is 0, indicating that miR-1 binds perfectly with some sites in this region.

### Investigation of UTR features

There are totally 30 features for the UTR-SVM and the feature selection process chooses 18 features as the optimal UTR features (Table 3). We summarize some the observations in the following.

**Table 3:** The optimal UTR feature set

	Feature name		Feature name
1	Top site score	10	No. of positive sites with 8mer_m1

2	Total positive score	11	top score with 8mer_m1

3	Positive site number	12	top score 7mer_m1

4	Max No. of positive sites within 100 nts	13	top score with 7mer_A1

5	Density of positive sites	14	top score with 6mer

6	No. of potential sites with 8mer_A1	15	top score without perfect seed

7	No. of positive sites with 8mer_A1	16	No. of potential sites with 7mer_A1

8	Top score with 8mer_A1	17	No. of postive sites with 7mer_A1

9	No. of potential sites with 8mer_m1	18	length of utr

#### UTR length is not a factor that influences miRNA target recognition

Among the three groups of UTR features, the length of the UTR is left out by feature selection, suggesting an ill correlation between the length of UTR and miRNA target recognition. The histograms of UTR length (Additional file 1 Figure 11) also reflects this finding.

#### The more accurate the sites are predicted, the more accurate the UTR prediction is

The top Site-SVM score is the most important feature in UTR-SVM, and the higher the site score of a candidate site is, the higher probability the 3'UTR is predicted to be a real target. This observation agrees with those of other algorithms [9,28], which all accept a 3'UTR to be the target if one potential site has a score more than the cut-off score. Moreover, the third top feature is the number of positive sites. Note that the positive sites are the candidate sites that are predicted to be true by the Site-SVM. The histograms of the number of positive sites (Additional file 1 Figure 13) reveal that more than 80% of the negative targets are not predicted to have positive sites by Site-SVM, which partially explains the good specificity achieved by our Site-SVM. Maximum number of positive sites within 100 nts also plays an important role in the UTR-SVM, which is consistent with the fact that the effectiveness of binding sites will be enhanced if they are close (Additional file 1 Figure 12). All the 5 features about 8mer_A1 are important features and ranked within the top 15 in the UTR-SVM features. As shown in Additional file 1 Figure 13, almost no false targets have 8mer_A1 and 8mer_m1 seed matches.

### Performance evaluation of SVMicrO

**Figure 4:**
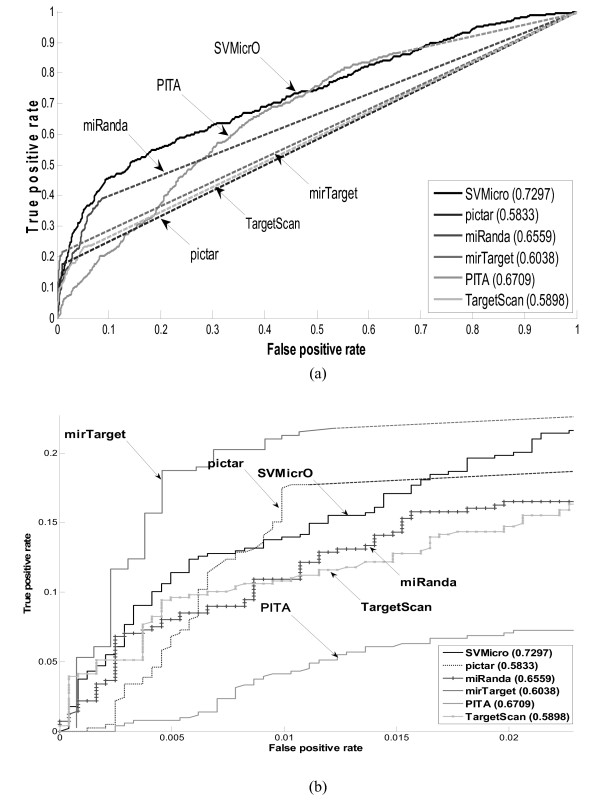
**Comparison of ROC curves based on training data**. *(a) Entire ROC curves based on training data (b) Zoom-in view of ROC curves based on training data *To investigate the performance of SVMicrO, the ROC performance was obtained from the cross-validation compared with several other popular target prediction algorithms including TargetScan, PITA, PicTar and miRanda.

**Figure 5:**
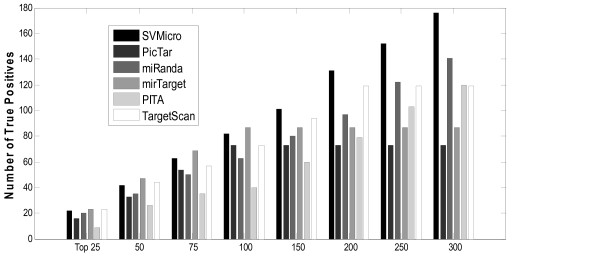
**Number of true positives among top ranked predictions**. This figure reveals the precision of each algorithm in terms of the number of true targets among the different numbers of top ranked genes.

To investigate the performance of SVMicrO, the ROC performance are obtained from the cross-validation compared with several other popular target prediction algorithms including TargetScan, PITA, PicTar and miRanda (Figure 4-(a)). Except PITA, for which predictions are obtained by the provided algorithm program, the TPR and FPR of the other algorithms were calculated based on the predictions published on their website. Notice that the curves for the compared algorithms are partially in broken lines at various TPR. This is because for TargetScan (v5.1), PicTar (2006), MirTarget2 (v3.0), PITA (v6), and miRanda (2008 Sept), the prediction scores for only a subset of mRNAs can be retrieved, while the scores of rest of mRNAs were assumed to be assigned by random predictors. There are two reasons for an mRNA to not receive a score. First, all these five existing algorithms apply different filters to remove unlikely sites/targets before proceeding to target prediction. mRNAs removed by the filter therefore receive no score and conceptually are predicted to be negative targets. Filtering does help reducing the search space for subsequent target prediction but at the price of reduced sensitivity; the reduction varies depending on the sensitivity of the filter employed by each algorithm. The filters of the four existing algorithms all rely heavily on the existence of the 6-mer seed match, and as discussed before, which result in around 20% reduction in sensitivity for the entire compared existing algorithms due to this filter. The no-score mRNAs for PITA are solely the result of the filter and it can be noted the sensitivity of the solid curve ends at around 80%. In fact, conceptually, the sensitivity of these algorithms should be capped at the sensitivity of the filter. In this case, assuming a random filter actually lends a performance advantage to these existing algorithms since it provides them a chance to increase beyond the capped sensitivity. In addition to the filter, TargetScan, PicTar and miRanda also apply a threshold to the prediction score to determine positive from negative. Since only positive targets are reported on the algorithm websites and we have neither access to the programs, nor the scores of the negative targets. Had the scores been available, the performance of the algorithm might be better or worse than a random predictor. However, we want to point out that regardless if these scores are available, SVMicrO will have the best sensitivity among the group since the filter designed for SVMicrO has only about 4% reduction in filtering step. Overall, SVMicrO has the largest AUC (Area-Under-the-Curve) and its ROC almost wraps around the curves of all other algorithms. Although PITA has the second largest AUC, it has the worst performance at low false positive rate. In the low False Positive Rate (FPR) region, the algorithms except PITA have similar performance for FPR < 0.01 while MirTarget has a slight edge over the rest. For 0.01 < FPR <0.3, SVMicrO clearly has the best and much larger sensitivity. At a practical FPR value of 0.1, SVMicrO increases the sensitivity about 6% over miRanda and at least 17% over MirTarget, TargetScan, and PicTar. Figure 4-(b) depicts the zoom-in view for FPR < 0.023. In this region, SVMicrO is only inferior to mirTarget. Although mirTarget has better TPR at low FPR, SVMicrO has much better sensitivity and obtains the best overall balance between TPR and FPR by achieving the largest AUC. To further reveal SVMicrO's performance at low FPR, we gauge the performance by evaluating the prediction precision. Precision represents the percentage of true targets among the predicted targets and can also be considered as the number of true targets among the given number of top ranked predictions. The precision of each algorithm in terms of the number of true targets among the different numbers of top ranked genes is revealed in Figure 5. For the top 100 predictions, SVMicrO and MirTarget produce very similar true positives and achieve better precisions than the other algorithms, while after top 150 predictions SVMicrO starts to set itself apart from the rest by achieving much higher precision. In summary, the validation based on training data indicates that SVMicrO attains the largest AUC, achieves highest TPR especially for low FPR, and has consistently better precisions; these results demonstrate clear performance improvement over many popular algorithms. SVMicrO's overall better performance was further validated next.

### Test on the proteomics data

**Figure 6:**
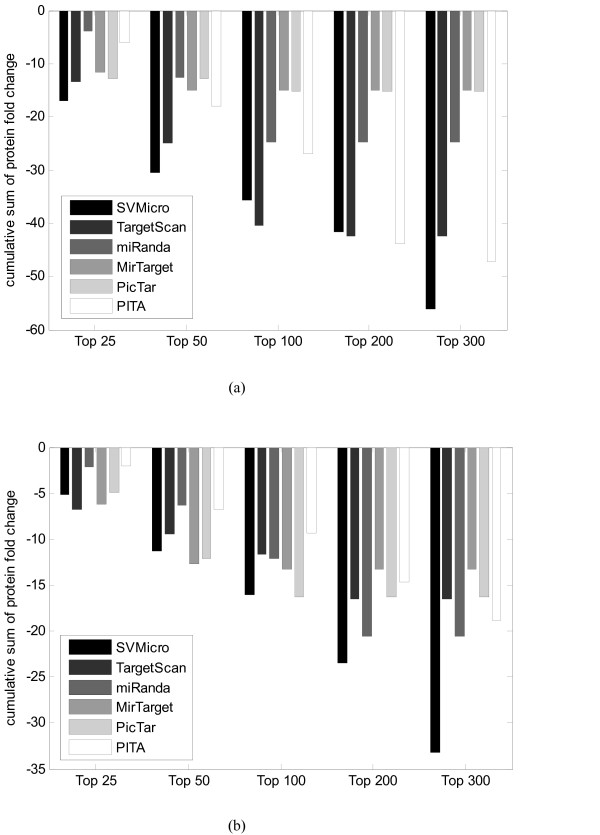
**Cumulative sum of protein fold change as a function of ranked predictions**. *(a)Cumulative sum of protein fold change as a function of ranked predictions of hsa-miR-124 (b) Cumulative sum of protein fold change as a function of ranked predictions of hsa-miR-1 *SVMicrO shows faster drop in CFC compare to other algorithm, which means SVMicrO achieves higher precision and smaller false positive.

To investigate the performance of SVMicrO on targets independent of the training data, we carried out the genome-wide target prediction for human miR-1, miR-16, miR-30a, miR-124, miR-155 and let-7b. Before prediction, the positive and negative targets of each miRNA were first removed from the training data and SVMicrO was retrained using the updated training data. The difficulty with validation is due to the lack of measurements of the ground truth. To mitigate the problem, we consulted the high throughput proteomics data in [29,30]. In these two papers, protein fold change due to the over-expression of specific miRNA were measured by stable-isotope-labelling-of-amino-acids-in culture (Additional file 1LAC) and quantified by LC/MS. Since protein inhibition is considered as primary mode of miRNA inhibition, protein down expression can be used as a utility for prediction validation. However, due to the limited coverage of LS/MS and relatively week intensity signals, no genes are declared targets definitively in the paper. Instead, it is only reasonable to assume that the larger down-fold a protein has, the more likely the corresponding gene is a true target. Due to the limitation of LC/MS coverage, only a subset of proteome is identified. Therefore, target prediction performance is only validated among these proteins. Figure 6 depicts the CFC (Cumulative Fold Change) for the top ranked 300 predictions of miR-124 and miR-1. Intuitively, CFC rewards higher confidence prediction with a drop and penalizes false prediction with a raise in the fold change. A better algorithm with higher precision and smaller false positive is expected to show faster drop in CFC. For miR-124, SVMicrO and TargetScan clearly set them apart from the rest, with SVMicrO performing slightly better up to top 100 and TargetScan having a slight edge up to top 200. At top 300, SVMicrO has clear advantage over the rest. For miR-1, SVMicrO is still among the better performing algorithms; instead of TargetScan, MirTarget and Pictar emerge to have competitive performance with SVMicrO. However, after top 200, SVMicrO achieves apparently much sharper drops than the others, compared with those of miR124. Moreover, same validation was carried also out for miR-16, miR-30a, miR-155 and let-7b and the significances of CFC prediction by each algorithm are assessed by random permutation (see Additional file 1 S.11).

Next, we further investigate the consistency of the prediction performance for each algorithm using the results of 6 miRNAs. A better algorithm should have a cumulative sum curve with two characteristics: 1) it drops faster at the beginning, signifying a higher precision, and 2) it has the highest overall drop. Therefore, we calculate the average area between the cumulative sum curve and the horizontal axis as a measurement of the performance for each algorithm

(1A) $$ℳ(n)=\frac{1}{n}\int_{0}^{n}c(x)dx$$

where *c*(*x*) denotes the function of cumulative sum curve and *n *denotes the number of the top ranked prediction. Intuitively, the smaller ℳ(*n*) is, the better the algorithm. Subsequently, a consistency measurement can be defined as the average value of ℳ(*n*) of the 6 miRNAs

(2A) $$C(n)=\sum_{i=1}^{6}{ℳ}_{i}(n).$$

The average area ℳ(*n*) of *n *∈ {20, 40, 80, 200} are calculated for SVMicrO, TargetScan, miRanda, MirTarget, PicTar as well as PITA. The result together with the rank (R) of each algorithm for miR124 and miR1 is shown in Table 4 and Table 5; they clearly shows SVMicrO is among the highest ranked algorithms at different *n*. The consistency measure C(n) was subsequently calculated and shown in Table 6. SVMicrO is the top ranked at all n. Based on these results, it is reasonable to conclude that SVMicrO is the most consistent algorithm that provides among the best prediction performance.

**Table 4:** Comparison of ℳ(*n*) and rank of hsa-miR-124

	SVMicrO		TargetScan		miRanda		MirTarget		PicTar		PITA	
**No. target**	**-**		**111**		**90**		**32**		**67**		**-**	

	**ℳ(*n*)**	**R**	**ℳ(*n*)**	**R**	**ℳ(*n*)**	**R**	**ℳ(*n*)**	**R**	**ℳ(*n*)**	**R**	**ℳ(*n*)**	**R**

Top 20	-7.59	1	-6.42	3	0.55	6	-5.53	5	-6.98	2	-5.80	4

Top 40	-12.90	1	-10.20	2	-2.58	6	-8.06	4	-9.40	3	-7.08	5

Top 80	-22.19	1	-19.06	2	-9.71	5	-8.06	6	-11.00	4	-12.94	3

Top 200	-32.38	1	-24.67	3	-11.30	4	-8.06	6	-11.00	5	-25.44	2

**Table 5:** Comparison of ℳ(*n*) and rank of hsa-miR-1

	SVMicrO		TargetScan		miRanda		MirTarget		PicTar		PITA	
**No. target**	**-**		**159**		**185**		**53**		**75**		**-**	

	**ℳ(*n*)**	**R**	**ℳ(*n*)**	**R**	**ℳ(*n*)**	**R**	**ℳ(*n*)**	**R**	**ℳ(*n*)**	**R**	**ℳ(*n*)**	**R**

Top 20	-3.05	2	-2.61	4	-0.51	6	-3.36	1	-2.76	3	-1.06	5

Top 40	-5.2	2	-4.99	3	-1.86	6	-5.98	1	-4.62	4	-2.33	5

Top 80	-8.49	2	-7.59	3	-4.62	6	-7.52	4	-8.99	1	-4.75	5

Top 200	-14.39	1	-10.56	3	-11.29	2	-7.52	6	-8.99	4	-7.66	5

**Table 6:** Comparison of consistency C(n) and rank

	SVMicrO		TargetScan		miRanda		MirTarget		PicTar		PITA	
**No. target**	**-**		**125**		**183**		**61**		**78**		**-**	

	C(n)	**R**	C(n)	**R**	C(n)	**R**	C(n)	**R**	C(n)	**R**	C(n)	**R**

Top 20	-5.03	1	-3.62	4	-2.03	5	-4.83	2	-3.7	3	-1.4	6

Top 40	-8.52	1	-6.48	3	-4.57	5	-7.63	2	-6.39	4	-2.2	6

Top 80	-14.17	1	-10.74	2	-9.47	5	-10.25	3	-9.5	4	-4.93	6

Top 200	-25.97	1	-16.24	2	-15.96	3	-11	5	-10.39	6	-11.35	4

### Test on the IP pull-down data

**Figure 7:**
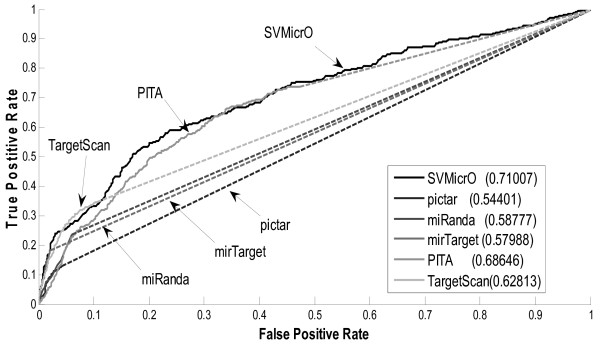
**ROC curves for the predictions of miR-124 tested on IP pull-downs**. The ROC curves were plotted based on 388 high confidence positive targets determined by IP pull down experiment.

**Figure 8:**
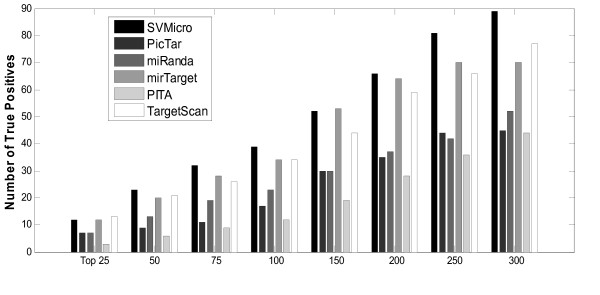
**Number of true positives among top ranked predictions of miR-124**.

**Figure 9:**
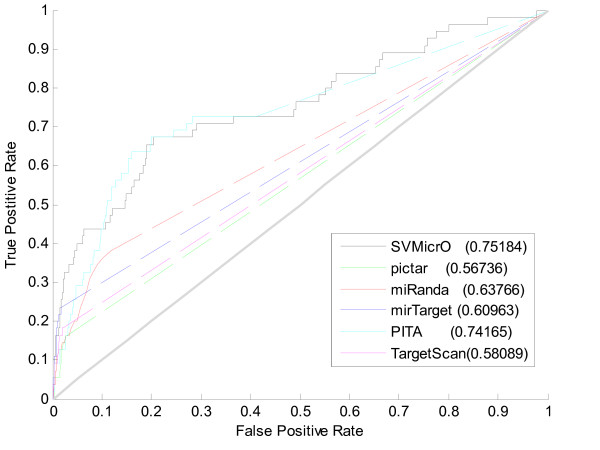
**ROC curves for the predictions of miR-1 tested on IP pull-downs**. The ROC curves were plotted based on 56 high confidence positive targets determined by IP pull down experiment.

**Figure 10:**
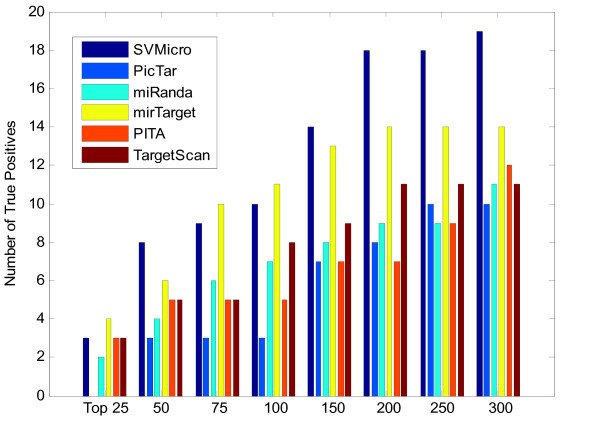
**Number of true positives among top ranked predictions of miR-1**.

Although the above experiments demonstrate consistently better performance achieved by SVMicrO, the utility of the evaluation on proteomic data might be limited by the coverage and potential noise in protein quantification. We therefore further validated the prediction of miR-124 and miR-1 on the IP pull-down data[31]. In these experiments, each miRNA was transfected in 293 cell and immunoprecipitation of the ARG-2 protein, an important component of the miRNA effector protein complexes, was carried out; the expression of genes recruited by ARG-2, or most likely the miRNA targets, was analyzed by microarray, and the target genes should be expressed in the microarray. 388 genes for miR-124 and 56 genes for miR-1 were determined in the end to be highly expressed at a stringent FDR level of 0.01. Although this technology has its own limitation, it nevertheless complements the proteomics data for prediction validation. Particularly, we treated the 388 and 56 highly expressed genes as the true targets of miR-124 and miR-1, respectively and investigated the ROC performance of different algorithms (Figure 7). Again, SVMicrO has the overall best performance supported by the largest AUC. Very similar phenomenon as the performance tested using the training data can be observed; in the low FPR region, SVMicrO, MirTarget, and TargetScan have similar sensitivity. But for FPR > 0.01 the existing algorithms cannot achieve satisfied TPR due mainly to the poor performance of their adopted filters. We further investigated the prediction precision (Figure 8). Clearly, SVMicrO attains the highest number of TPs for all the tested numbers of top ranked predictions, and thus has the best prediction precision. Figure 7 and 8 reflect the similar performance improvement of SVMicrO over other algorithms as that demonstrated by the proteomics data. Same tests were also carried out for miR-1 (Figure 9 and 10). Again, similar conclusion can be drawn from these figures, which reinstate the consistent higher performance achieved by SVMicrO. In contrast, other algorithms do not show similar consistency; unlike the case of miR-124, TargetScan has worse performance than miRanda this time. Based on these results, we can conclude confidently that SVMicrO achieves improved prediction sensitivity, specificity, and precision than the existing algorithms.

## Discussion

The improved performance of SVMicrO can be attributed to the following three factors. First, a comprehensive training data set including a large number of verified positive targets and derived negative targets for a diverse group of miRNAs was constructed. Compared with the training data constructed for other existing data driven algorithms, this training set contains by far the largest number of verified targets. As a result, this training set possesses a better coverage of different characteristics of miRNA target recognition than any other existing training datasets. Secondly, due to the increased size of training data, we could afford to develop more sophisticated prediction algorithms to better uncover the important targeting characteristics from data. SVMicrO algorithm has a unique two-stage structure, where the miRNA binding sites are first predicted, which is then followed by the prediction of the 3'UTR in the second stage. In each stage, the prediction is accomplished by a SVM algorithm. The performance improvement can be considered as a result of the sophisticated SVM algorithm to properly model not only binding sites but especially their relationship with 3'UTR. Thirdly, the improved performance is also an outcome of the site and UTR feature sets that, when combined, encompasses the largest extraction of features, surveying extensive characteristics of miRNA target binding. In addition, the adopted feature selection algorithm also ensures that only the optimal set of features is chosen for target prediction; this feature selection not only increases the computational efficiency by removing the correlated features but also ensures the best performance by eliminating the potential distortion and noise introduced by the non-effective features.

Even though SVMicrO achieves the improved performance, it is evident from the evaluation that further improvement is needed. For a data driven algorithm, further improvement comes at the expense of increased quality and quantity of training data set. Compared with the number of potential genome-wide miRNA targets, our training data set is still relatively small in size and thus cannot cover all features of miRNA binding. Moreover, collecting representative negative targets is also a challenge. On the one hand, there is almost no reported, verified negative target. On the other hand, the number of negative targets is much larger than that of the positive targets, making the training data highly imbalanced. This created enormous difficulty for computational algorithms to learn the features of true targets. Improving the quality of training data especially the negative targets will be an important future research topic. Since SVMicrO is shown to be able to achieve robust performance on the current training data, it holds the promise to achieve continuing improvement whenever better training data that contain additional verified or high confidence positive targets and properly selected negative targets are available.

## Conclusions

We proposed in this paper a new data driven algorithm, SVMicrO, for prediction of mammalian miRNA targets. Comprehensive validation of SVMicrO using a large training data set, the proteomics, and the IP pull down data has confirmed that SVMicrO can produce consistently better sensitivity, specificity, and precision than several popular existing algorithms. Genome-wide prediction of human miRNA using SVMicrO has been carried out. All the related materials including source code of SVMicrO and generation of miRNA binding logo and prediction results are available at http://compgenomics.utsa.edu/svmicro.html.

## Authors' contributions

HL, SJG, and YH conceived the idea. HL, YC, YH worked out the detailed derivations. HL and DY implemented the algorithm and performed the prediction. HL, DY, YH wrote the paper. All authors read and approved the final manuscript.

## Supplementary Material

Additional file 1 **Supplementary Information**. Click here for file
