# Supplementary material for: Improving performance of mammalian microRNA target prediction
Source: BMC Bioinformatics. 2010 Sep 22;11:476. doi: 10.1186/1471-2105-11-476 (PMC2955701; doi:10.1186/1471-2105-11-476)
Supplement: Additional file 1 — Supplementary Information. [file 1471-2105-11-476-S1.PDF]

SUPPLEMENTARY INFORMATION

Improving Performance of Mammalian MicroRNA Target Prediction

Hui Liu[1], Dong Yue[2], Yidong Chen[4,5], Shou-Jiang Gao[3,5] and Yufei Huang[2,5*]

[1]SIEE, China University of Mining and Technology, Xuzhou, Jiangsu, CHINA.

[2]Department of ECE, University of Texas at San Antonio, [3] Department of Pediatrics, [4] Department of Epidemiology and Biostatistics, [5]Greehey Children's Cancer Research Institute, University of Texas Health Science Center at San Antonio.

S.1. SENISITIVTIES OF THE PROPOSED POTENTIAL SITE FILTER

Table 1. Sensitivities of the proposed filter and the rule based on 6-mer seed match obtained on training data.

Seed Match Rules	Sensitivity of Site Detection	Sensitivity of UTR Detection
6mer perfect match	79.8%	77.1%
proposed rules	96.2	95.8%

S.2. DETAILS ABOUT CONSERVATION SCORES

The conservation score is calculated using the phastCons28way table from the UCSC genome browser. The phastCons28way table is produced by the phastCons program based on a whole-genome alignment of 28 species. The detail of phastCons28way table is as follow:

```
ALPHABET: A C G T
ORDER: 0
SUBST_MOD: REV
BACKGROUND: 0.295000 0.205000 0.205000 0.295000
RATE_MAT:
  -0.990634    0.178819    0.490738    0.321078
   0.257325   -1.001677    0.186563    0.557790
   0.706184    0.186563   -1.161873    0.269126
   0.321078    0.387615    0.187019   -0.895713
TREE:
((((((((((((hg18:0.005873,panTro2:0.007668):0.026074,rheMac2:0.031973):0.073300,otoGar1:0.151185):0.015682,tupBel1:0.162844):0.006272,(((rn4:0.084383,mm8:
0.076274):0.200607,cavPor2:0.202990):0.034350,oryCun1:0.208548):0.014587):0.019763,((sorAra1:0.248532,eriEur1:0.222255):0.045693,(((canFam2:0.101137,fel
Cat3:0.098203):0.048213,equCab1:0.099323):0.007287,bosTau3:0.163945):0.012398):0.018928):0.030081,(dasNov1:0.133274,(loxAfr1:0.103030,echTel1:0.232706)
:0.049511):0.008424):0.213469,monDom4:0.320721):0.088647,ornAna1:0.488110):0.118797,(galGal3:0.395136,anoCar1:0.513962):0.093688):0.151358,xenTro2:0.7
78272):0.174596,(((tetNig1:0.203933,fr2:0.239587):0.203949,(gasAcu1:0.314162,oryLat1:0.501915):0.055354):0.346008,danRer4:0.730028):0.174596);
```

S.3. BRIEF SUMMARY OF SITE FEATURES

Table 2. Brief summary of all site features.

Index	Feature name	Data type	Group	Explanation	Typical value	
					Min	Max
1	consv_3cntxt	FLOAT	conservation	seed's 3' context conservation score	0	1
2	consv_seed	FLOAT	conservation	seed conservation score	0	1
3	consv_5cntxt	FLOAT	conservation	seed's 5' context conservation score	0	1
4	sm_6mer	INTEGER	seed match type	6mer seed match	0	1
5	sm_7mer_A1	INTEGER	seed match type	7mer_A1 seed match	0	1
6	sm_7mer_m1	INTEGER	seed match type	7mer_m1 seed match	0	1
7	sm_7mer_m8	INTEGER	seed match type	7mer_m8 seed match	0	1
8	sm_8mer_A1	INTEGER	seed match type	8mer_A1 seed match	0	1
9	sm_8mer_m1	INTEGER	seed match type	8mer_m1 seed match	0	1
10	to_stop_codon	INTEGER	position	distance to stop codon	0	2000
11	to_ends	INTEGER	position	distance to nearest end	0	2000
12	ratio_to_ends	FLOAT	position	ratio to nearest end	0	1
13	nt1	INTEGER	nt match status	p1 match status	1	4
14	nt2	INTEGER	nt match status	p2 match status	1	4
15	nt3	INTEGER	nt match status	p3 match status	1	4
16	nt4	INTEGER	nt match status	p4 match status	1	4
17	nt5	INTEGER	nt match status	p5 match status	1	4
18	nt6	INTEGER	nt match status	p6 match status	1	4
19	nt7	INTEGER	nt match status	p7 match status	1	4
20	nt8	INTEGER	nt match status	p8 match status	1	4
21	nt9	INTEGER	nt match status	p9 match status	1	4
22	nt10	INTEGER	nt match status	p10 match status	1	4
23	nt11	INTEGER	nt match status	p11 match status	1	4
24	nt12	INTEGER	nt match status	p12 match status	1	4
25	nt13	INTEGER	nt match status	p13 match status	1	4
26	nt14	INTEGER	nt match status	p14 match status	1	4
27	nt15	INTEGER	nt match status	p15 match status	1	4
28	nt16	INTEGER	nt match status	p16 match status	1	4
29	nt17	INTEGER	nt match status	p17 match status	1	4
30	nt18	INTEGER	nt match status	p18 match status	1	4

31	nt19	INTEGER	nt match status	p19 match status	1	4
32	nt20	INTEGER	nt match status	p20 match status	1	4
33	2mer1	INTEGER	2mer match status	2mer1 match status	1	16
34	2mer2	INTEGER	2mer match status	2mer2 match status	1	16
35	2mer3	INTEGER	2mer match status	2mer3 match status	1	16
36	2mer4	INTEGER	2mer match status	2mer4 match status	1	16
37	2mer5	INTEGER	2mer match status	2mer5 match status	1	16
38	2mer6	INTEGER	2mer match status	2mer6 match status	1	16
39	2mer7	INTEGER	2mer match status	2mer7 match status	1	16
40	2mer8	INTEGER	2mer match status	2mer8 match status	1	16
41	2mer9	INTEGER	2mer match status	2mer9 match status	1	16
42	2mer10	INTEGER	2mer match status	2mer10 match status	1	16
43	2mer11	INTEGER	2mer match status	2mer11 match status	1	16
44	2mer12	INTEGER	2mer match status	2mer12 match status	1	16
45	2mer13	INTEGER	2mer match status	2mer13 match status	1	16
46	2mer14	INTEGER	2mer match status	2mer14 match status	1	16
47	2mer15	INTEGER	2mer match status	2mer15 match status	1	16
48	2mer16	INTEGER	2mer match status	2mer16 match status	1	16
49	2mer17	INTEGER	2mer match status	2mer17 match status	1	16
50	2mer18	INTEGER	2mer match status	2mer18 match status	1	16
51	2mer19	INTEGER	2mer match status	2mer19 match status	1	16
52	rgs_match	INTEGER	region	number of match in seed region	0	8
53	rgs_gu	INTEGER	region	number of mismatch in seed region	0	8
54	rgs_mismatch	INTEGER	region	number of G:U in seed region	0	8
55	rgs_gap	INTEGER	region	number of gap in seed region	0	8
56	rgs_bulge	INTEGER	region	number of bulge in seed region	0	2
57	rgs_bulge_nt	INTEGER	region	number of bulged nts in seed region	0	2
58	rgs_energy	FLOAT	region	binding energy of seed region	-10	5
59	rg3_match	INTEGER	region	number of match in 3' region	0	8
60	rg3_gu	INTEGER	region	number of mismatch in 3' region	0	8
61	rg3_mismatch	INTEGER	region	number of G:U in 3' region	0	8
62	rg3_gap	INTEGER	region	number of gap in 3' region	0	8
63	rg3_bulge	INTEGER	region	number of bulge in 3' region	0	2
64	rg3_bulge_nt	INTEGER	region	number of bulged nts in 3' region	0	5
65	rg3_energy	FLOAT	region	binding energy of 3' region	-10	5
66	rgt_match	INTEGER	region	number of match in total region	0	15
67	rgt_gu	INTEGER	region	number of mismatch in total region	0	15
68	rgt_mismatch	INTEGER	region	number of G:U in total region	0	15
69	rgt_gap	INTEGER	region	number of gap in total region	0	15
70	rgt_bulge	INTEGER	region	number of bulge in total region	0	4
71	rgt_bulge_nt	INTEGER	region	number of bulged nts in total region	0	12
72	rgt_energy	FLOAT	region	binding energy of total region	-20	10
73	acc_energy	FLOAT	accessbility energy	accessbility energy	-20	10
74	cntxt_A_cntnt	FLOAT	context	A content in context	0	1
75	cntxt_C_cntnt	FLOAT	context	C content in context	0	1
76	cntxt_G_cntnt	FLOAT	context	G content in context	0	1
77	cntxt_U_cntnt	FLOAT	context	U content in context	0	1
78	cntxt_AA_cntnt	FLOAT	context	AA content in context	0	1
79	cntxt_AC_cntnt	FLOAT	context	AC content in context	0	1
80	cntxt_AG_cntnt	FLOAT	context	AG content in context	0	1
81	cntxt_AU_cntnt	FLOAT	context	AU content in context	0	1
82	cntxt_CA_cntnt	FLOAT	context	CA content in context	0	1
83	cntxt_CC_cntnt	FLOAT	context	CC content in context	0	1
84	cntxt_CG_cntnt	FLOAT	context	CG content in context	0	1
85	cntxt_CU_cntnt	FLOAT	context	CU content in context	0	1
86	cntxt_GA_cntnt	FLOAT	context	GA content in context	0	1
87	cntxt_GC_cntnt	FLOAT	context	GC content in context	0	1
88	cntxt_GG_cntnt	FLOAT	context	GG content in context	0	1
89	cntxt_GU_cntnt	FLOAT	context	GU content in context	0	1
90	cntxt_UA_cntnt	FLOAT	context	UA content in context	0	1
91	cntxt_UC_cntnt	FLOAT	context	UC content in context	0	1
92	cntxt_UG_cntnt	FLOAT	context	UG content in context	0	1
93	cntxt_UU_cntnt	FLOAT	context	UU content in context	0	1
94	cntxt_pos_n8	INTEGER	context	nt type of -8	1	4

95	cntxt_pos_n7	INTEGER	context	nt type of -7	1	4
96	cntxt_pos_n6	INTEGER	context	nt type of -6	1	4
97	cntxt_pos_n5	INTEGER	context	nt type of -5	1	4
98	cntxt_pos_n4	INTEGER	context	nt type of -4	1	4
99	cntxt_pos_n3	INTEGER	context	nt type of -3	1	4
100	cntxt_pos_n2	INTEGER	context	nt type of -2	1	4
101	cntxt_pos_n1	INTEGER	context	nt type of -1	1	4
102	cntxt_pos_n0	INTEGER	context	nt type of -0	1	4
103	cntxt_pos_p1	INTEGER	context	nt type of +1	1	4
104	cntxt_pos_r1	INTEGER	context	nt type of r1	0	4
105	cntxt_pos_r2	INTEGER	context	nt type of r2	0	4
106	cntxt_pos_r3	INTEGER	context	nt type of r3	0	4
107	cntxt_pos_r4	INTEGER	context	nt type of r4	0	4
108	cntxt_pos_r5	INTEGER	context	nt type of r5	0	4
109	cntxt_pos_r6	INTEGER	context	nt type of r6	0	4
110	cntxt_pos_r7	INTEGER	context	nt type of r7	0	4
111	cntxt_pos_r8	INTEGER	context	nt type of r8	0	4
112	cntxt_pos_r9	INTEGER	context	nt type of r9	0	4
113	cntxt_pos_r10	INTEGER	context	nt type of r10	0	4

S.4. BRIEF SUMMARY OF UTR FEATURES

Table 3. Brief summary of all UTR features.

Index	Feature name	Data type	Group	Explanation	Typical value	
					Min	Max
1	utr_len	INTEGER	utr length	length of utr	0	2000
2	psite_dens	FLOAT	density	density of potential site in entire UTR	0	0.1
3	max_partial_psite_num	INTEGER	density	max number of potential site in 100 nt	0	5
4	pos_site_dens	FLOAT	density	density of positive site in entire UTR	0	0.01
5	max_partial_pos_site_num	INTEGER	density	max number of positive site in 100 nt	0	2
6	total_pos_score	FLOAT	globe site score	total score of positive sites	-2	2
7	psite_num	INTEGER	globe site score	number of potential sites	0	50
8	pos_site_num	INTEGER	globe site score	number of postive sites	0	5
9	top_score	FLOAT	globe site score	top score of all potential sites	-2	2
10	psite_num_6mer	INTEGER	site score of seed type	number of potential sites with 6mer seed	0	2
11	pos_site_num_6mer	INTEGER	site score of seed type	number of postive sites with 6mer seed	0	2
12	top_score_6mer	FLOAT	site score of seed type	top score of all potential sites with 6mer seed	-2	2
13	psite_num_7mer_A1	INTEGER	site score of seed type	number of potential sites with 7mer_A1 seed	0	2
14	pos_site_num_7mer_A1	INTEGER	site score of seed type	number of postive sites with 7mer_A1 seed	0	2
15	top_score_7mer_A1	FLOAT	site score of seed type	top score of all potential sites with 7mer_A1 seed	-2	2
16	psite_num_7mer_m1	INTEGER	site score of seed type	number of potential sites with 7mer_m1 seed	0	2
17	pos_site_num_7mer_m1	INTEGER	site score of seed type	number of postive sites with 7mer_m1 seed	0	2
18	top_score_7mer_m1	FLOAT	site score of seed type	top score of all potential sites with 7mer_m1 seed	-2	2
19	psite_num_7mer_m8	INTEGER	site score of seed type	number of potential sites with 7mer_m8 seed	0	2
20	pos_site_num_7mer_m8	INTEGER	site score of seed type	number of postive sites with 7mer_m8 seed	0	2
21	top_score_7mer_m8	FLOAT	site score of seed type	top score of all potential sites with 7mer_m8 seed	-2	2
22	psite_num_8mer_A1	INTEGER	site score of seed type	number of potential sites with 8mer_A1 seed	0	2
23	pos_site_num_8mer_A1	INTEGER	site score of seed type	number of postive sites with 8mer_A1 seed	0	2
24	top_score_8mer_A1	FLOAT	site score of seed type	top score of all potential sites with 8mer_A1 seed	-2	2
25	psite_num_8mer_m1	INTEGER	site score of seed type	number of potential sites with 8mer_m1 seed	0	2
26	pos_site_num_8mer_m1	INTEGER	site score of seed type	number of postive sites with 8mer_m1 seed	0	2
27	top_score_8mer_m1	FLOAT	site score of seed type	top score of all potential sites with 8mer_m1 seed	-2	2
28	psite_num_other	INTEGER	site score of seed type	number of potential sites without perfect seed	0	2
29	pos_site_num_other	INTEGER	site score of seed type	number of postive sites without perfect seed	0	2
30	top_score_other	FLOAT	site score of seed type	top score of all potential sites without perfect seed	-2	2

S.5. GENERATION OF NEGATIVE TRAINING DATA

The negative training targets are generated using microarray data of miRNA transfection. To cover the binding feature variations due to different miRNAs, microarray data for 20 different miRNAs, each also including time series measurements were download from Gene Expression Omnibus (GEO) (SI Table 4). To select high quality negative targets, we carefully designed a set of selection criterions. First, the differential expression *p* value of the negative target must be less than 0.001 to ensure it is differentially expressed. Second, the fold change (FC) of the negative target must be greater than 1.5 to ensure it is not down-regulated. To determine the FC threshold value 1.5, we first collected the FCs of the verified targets in our training data from these microarray experiments, from which expression FCs of 209 positive targets are acquired. The histogram is shown in Figure 1. As expected, the true targets are likely to be down-regulated at mRNA level; however, a few genes are also up-regulated, due to possibly either the secondary effect of miRNA repression or miRNA solely inhibiting translation of these genes. In Figure 1, notice that fold changes of all these positive genes are less than 1. As a comparison, we also examined the distribution of genome-wide mRNA expression after miRNA transfection. An example of mir-124 is shown in Figure 2. We observe that the distribution of genome-wide expression FC is roughly symmetric about 0; an implication is that selecting positive target based solely on mRNA downregulation would produce a larger number of false positives. A more relevant indication is that the significantly up-regulated genes, say fold change > 1.5 are mostly negative targets. In light of these observations, we choose the FC threshold to be 1.5. Thirdly, we also utilized the time series data and require that the first two tests must be satisfied at both 12h and 24h for a gene to be a negative target. SI Table 4 also shows the number of selected negative targets for each of the 20 microarray data; this number ranges from 4 to 676. Note that since microarray data cannot reveal translationally repressed targets, some of the upregulated genes in microarray could still be the true targets. Therefore, the negative training data could contain false negatives, although the number should be small due to the stringent selection criterions.

Table 4. Data Source for Negative Samples.

Index	miRNA	GEO Dataset ID	NO. of negative sample
1	hsa-let-7c	GSM156557, GSM156558	29
2	hsa-miR-15a	GSM156545, GSM156549	613
3	hsa-miR-16	GSM156546, GSM156550	587
4	hsa-miR-17	GSM156553, GSM156555	115
5	hsa-miR-192	GSM156547, GSM156551	77
6	hsa-miR-20a	GSM156554, GSM156556	108
7	hsa-miR-215	GSM156548, GSM156552	92
8	has-miR-192	GSM328290, GSM328287	21
9	has-mirR-215	GSM328291, GSM328288	20
10	hsa-miR-122	GSM210900, GSM210901	13
11	hsa-miR-128	GSM210902, GSM210903	10
12	hsa-miR-132	GSM210904, GSM210905	11
13	hsa-miR-133a	GSM210906, GSM210907	203
14	hsa-miR-142-3p	GSM210908, GSM210909	38
15	hsa-miR-148b	GSM210910, GSM210911	42
16	hsa-miR-34a	GSM187633, GSM187634, GSM187631, GSM187632	676
17	hsa-miR-34b	GSM190765, GSM190757	424
18	hsa-miR-34c-5p	GSM190758, GSM190766	451
19	hsa-miR-7	GSM210896, GSM210897	8
20	hsa-miR-9	GSM210898, GSM210899	4
**Total No.**			3542 (3492 pairs left after removing reduplicates)

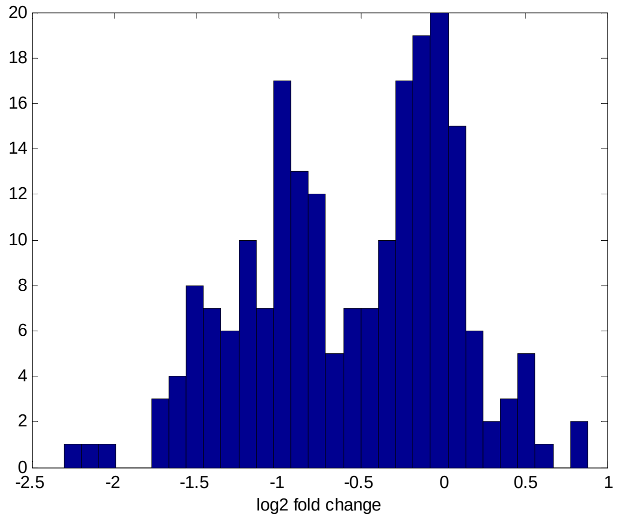

Fig 1. Fold change distribution of (209) positive targets from miRecords.

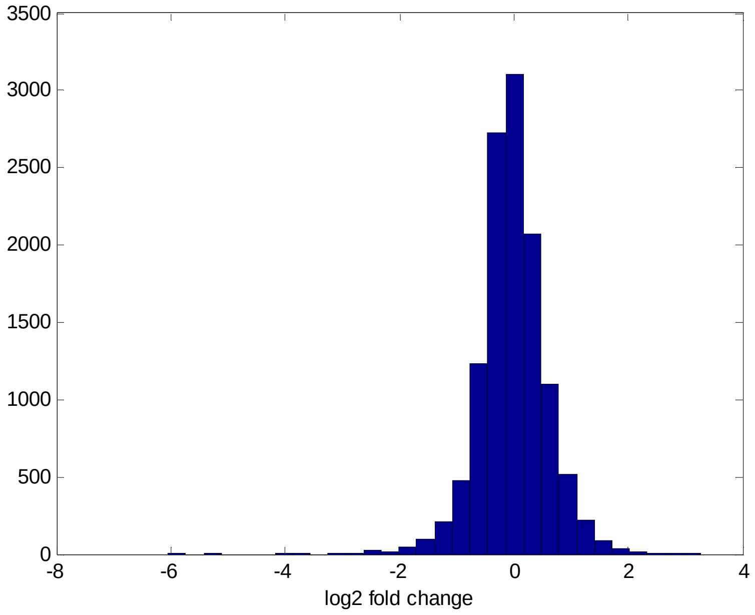

Fig 2. Genome-wide fold change distribution of mir-124 transfection. Data is obtained from [30].

S.6. HISTOGRAMS OF FEATURES

The independent empirical distributions of each site feature in the forms of histograms were obtained from the positive and negative data. Although, they do not reveal combinatory discriminative power of the features, they do provide information regarding the importance of the features in prediction. Particularly, if the distributions of a feature in the positive and negative target sites are similar, it means that the positive and negative target sites cannot be easily separated by this feature, and thus this feature bears low discriminative power, or in other word, is unlikely to be a good feature.

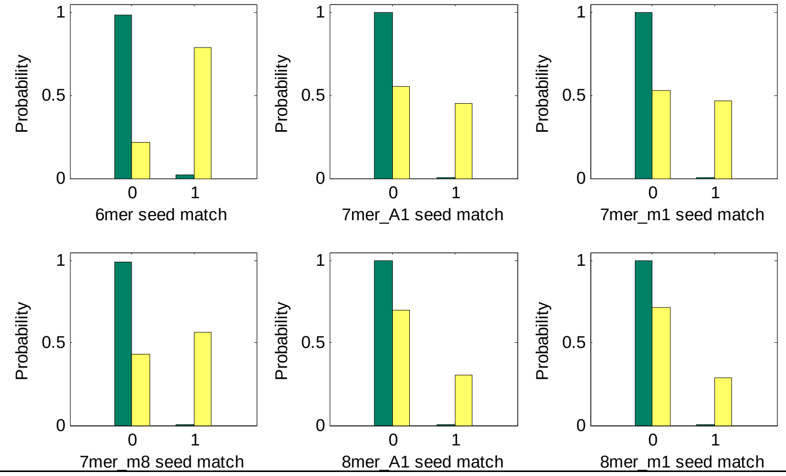

Fig. 3. Histograms of perfect seed match features.

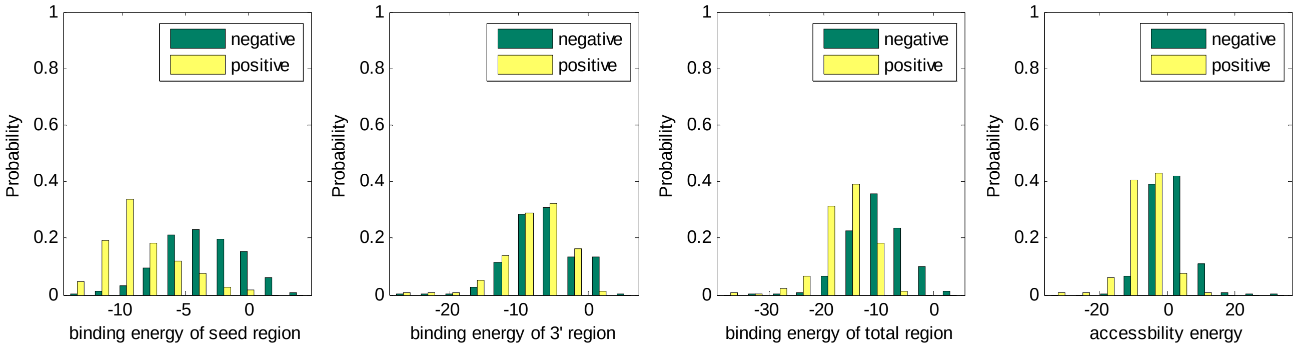

Fig. 4. Histograms of energy features**.**

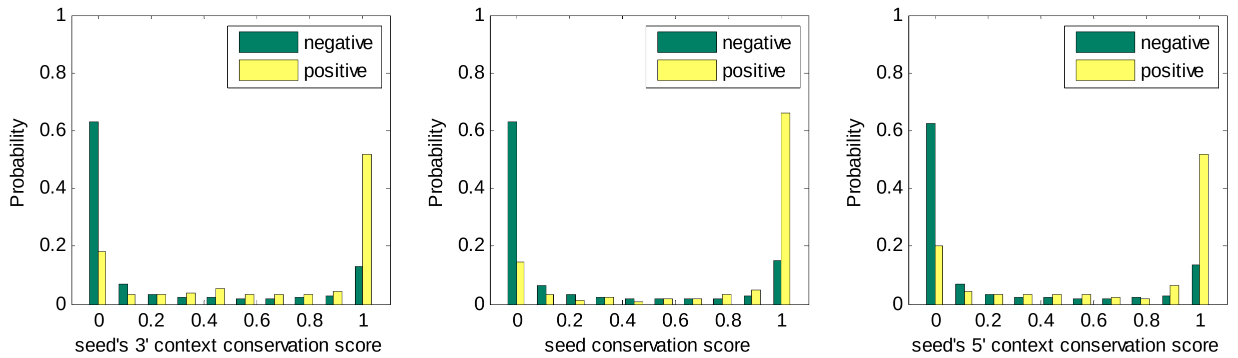

Fig. 5. Histograms of conservation features.

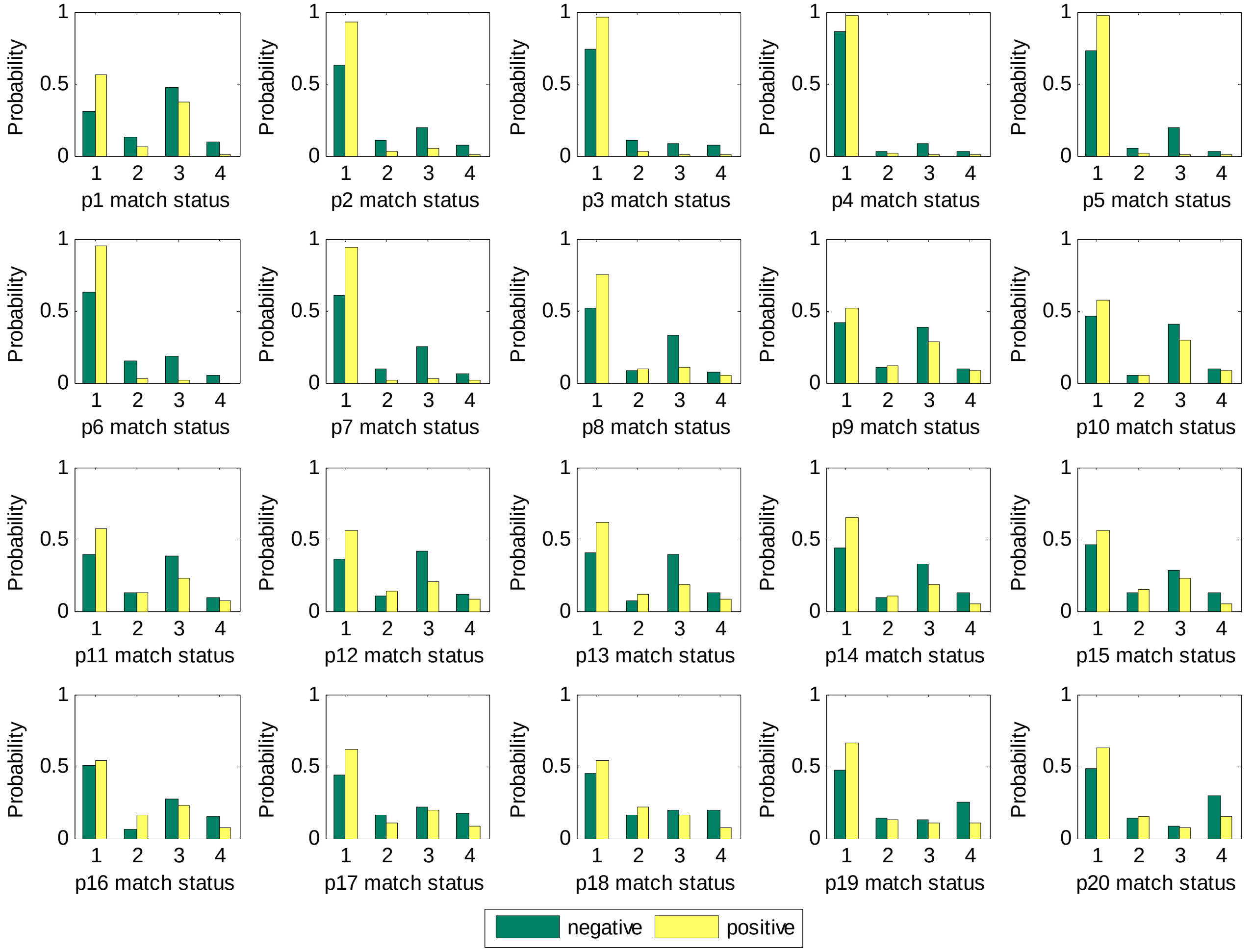

Fig. 6. Histograms of nt match features.

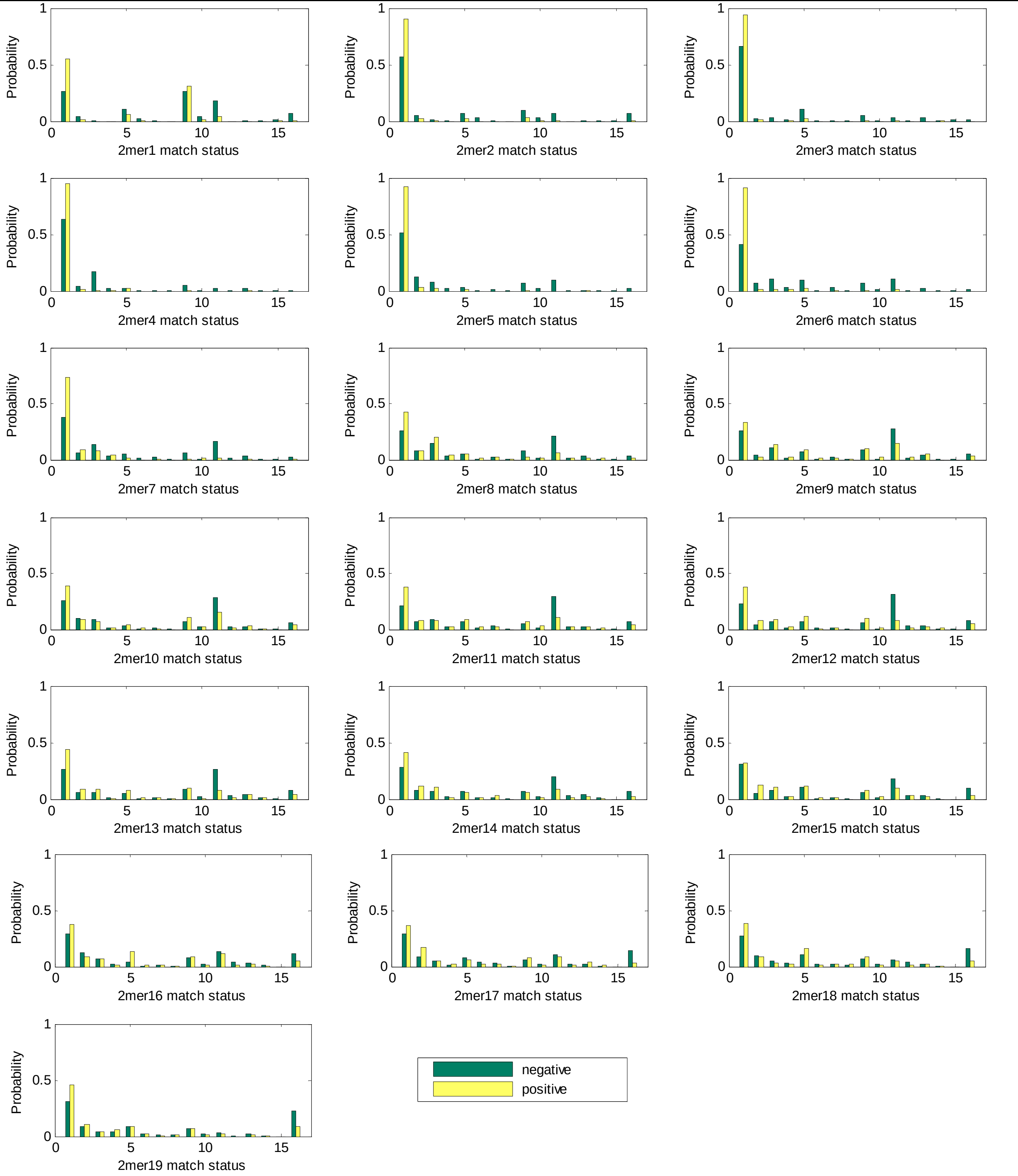

Fig. 7. Histograms of 2mer match features.

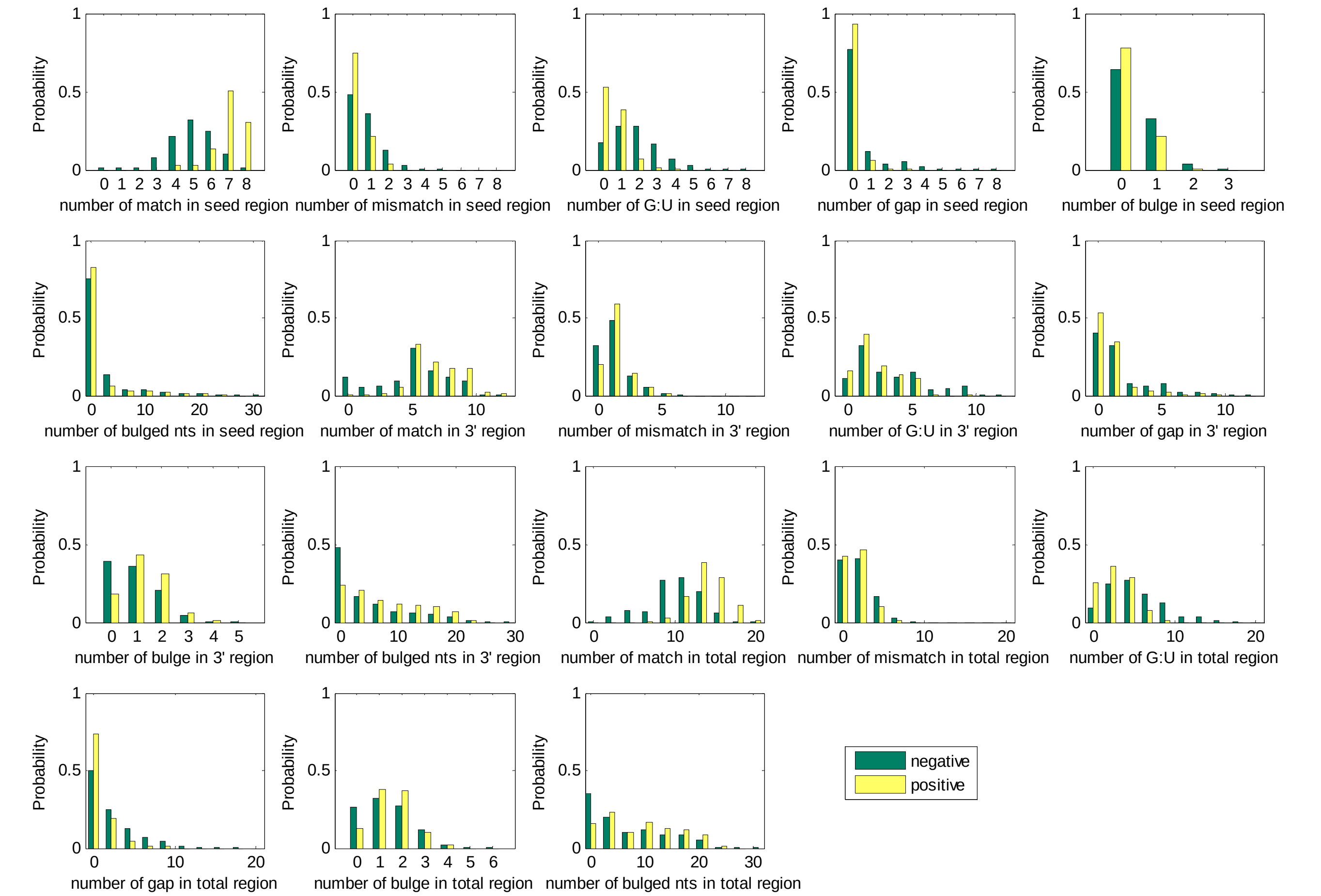

Fig. 8. Histogram of regional binding structure features.

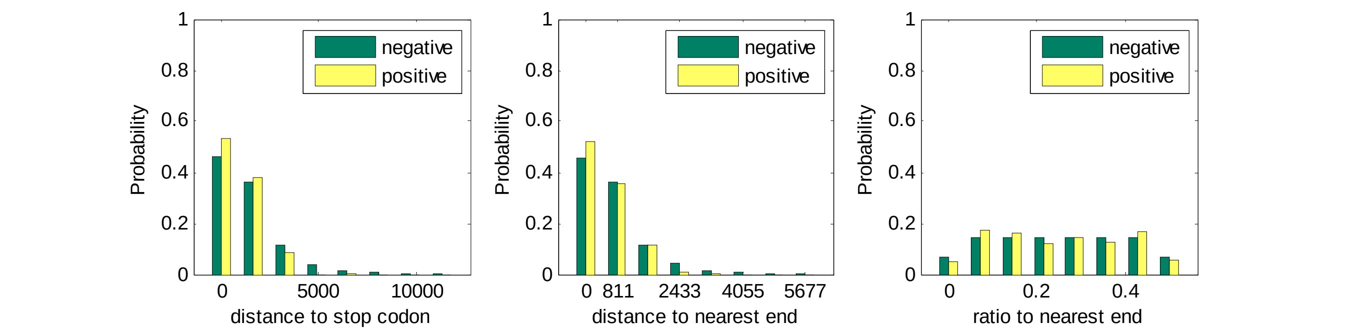

Fig. 9. Histogram of Position Features.

S.7 ROC PERFORMANCE OF SITE-SVM

To investigate the performance of Site-SVM, the receiver operating characteristic (ROC) performance is obtained from the cross-validation (Figure 10) based on the training dataset. The ROC evaluates the performance of the true positive rate (TPR), or sensitivity vs. the false positive rate (FPR), or 1-specificity. TPR denotes the chance of having predicted the entire true targets, while FPR measures the odds of falsely predicting a target. A better algorithm should have smaller FPR at a given TPR. In Figure 10, Site-SVM shows a better performance comparing to 6 types of perfect seed match. Moreover, Site-SVM presents a continuous curve, which means Site-SVM can calculate the confidence of a potential site to be a positive site, and this is meaningful for sequential identification work.

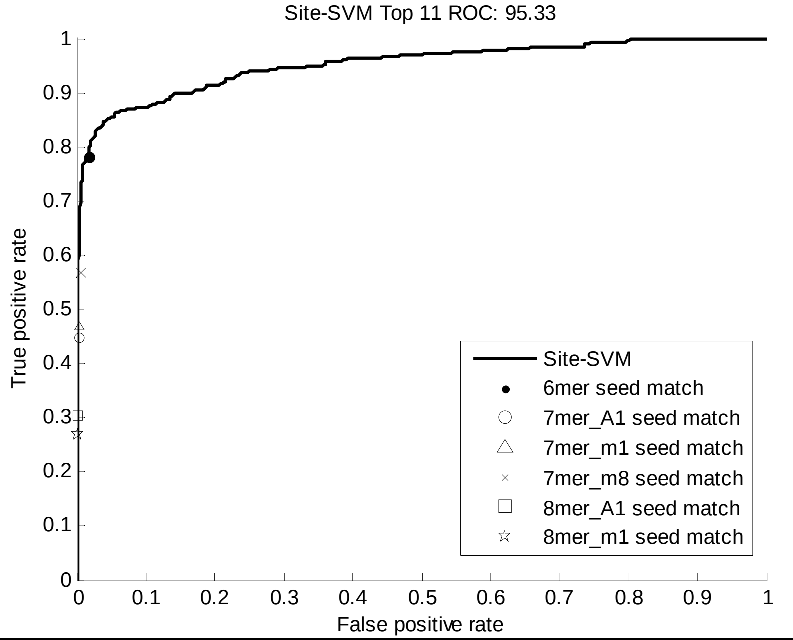

Fig. 10. ROC curve of Site-SVM and perfect seed match.

S.8. HISTOGRAMS OF UTR FEATURES

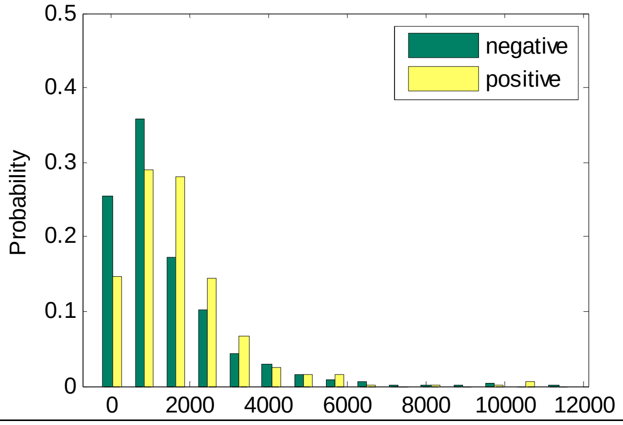

Fig. 11. Histogram of UTR Length Feature.

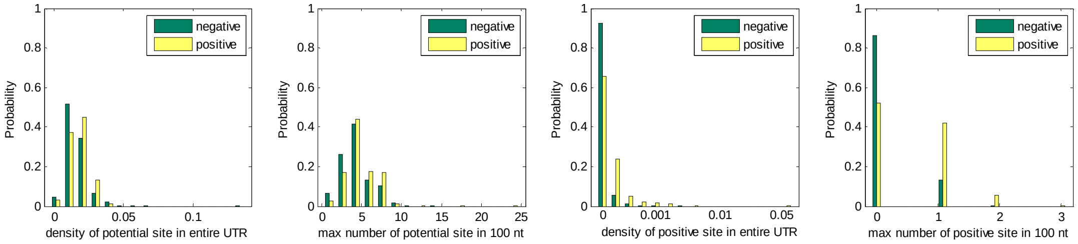

Fig. 12. Histograms of sites density features**.**

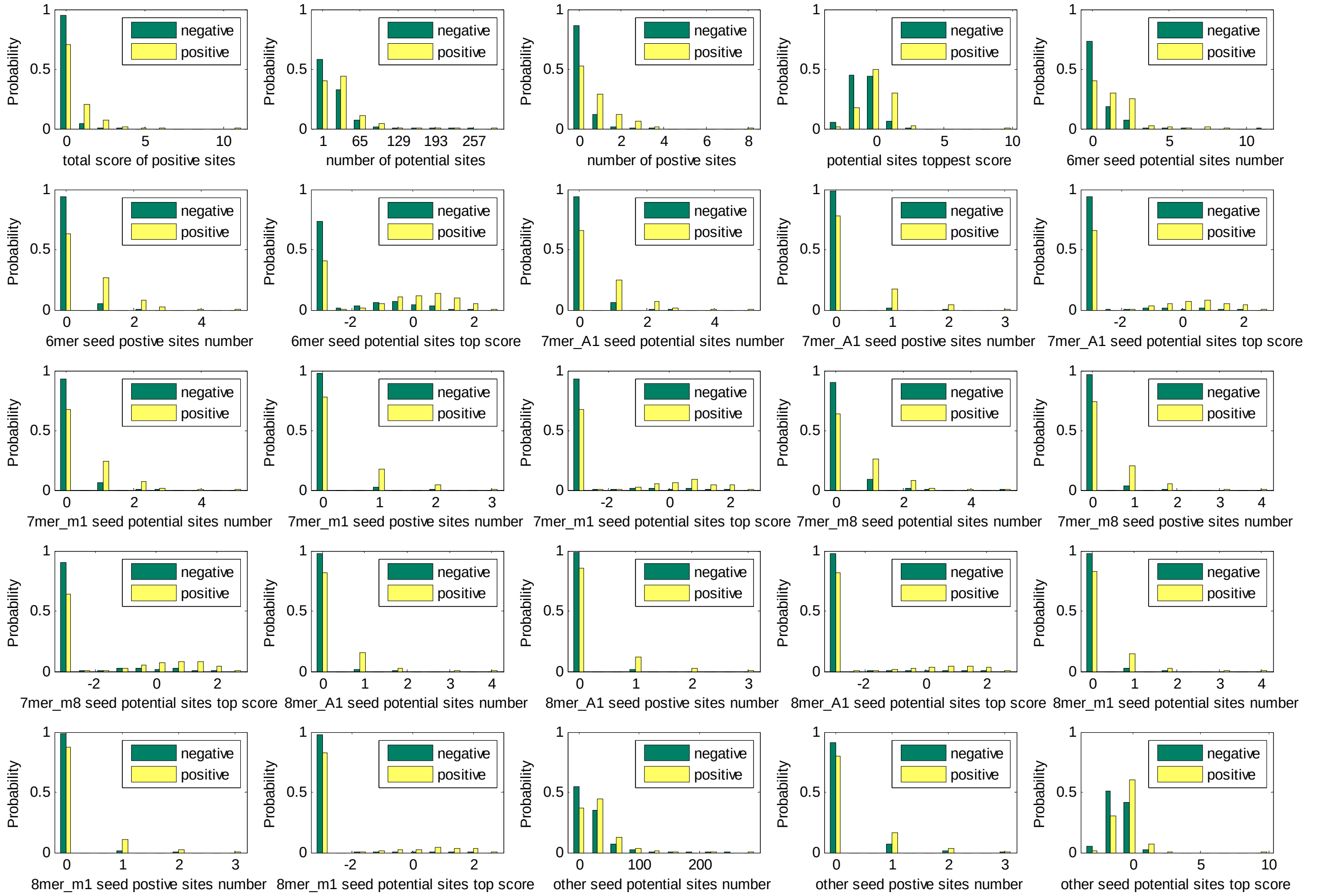

Fig. 13. Histograms of Sites Score Features.

S.9. STATISTICAL TESTS ON FEATURES

To quantify the independent discriminative power of each feature based on the histogram, three tests, i.e., Kullback-Leibler divergence, Jensen-Shannon divergence, and Chi-square test are applied to the empirical distributions of each feature. Each test can measure the difference between the distributions of a feature in the positive data *P* and in the negative data *N*. In our case, Jensen-Shannon divergence is more meaningful since it is a symmetric measure of the difference between two distributions while Kullback-Leibler divergence is a non-symmetric measure. The definitions of Kullback-Leibler divergence and Jensen-Shannon divergence are shown in (1) and (2), respectively

$$D_{KL}(P||N) = \int_{-\infty}^{+\infty} p(x) \log \frac{p(x)}{n(x)} dx \quad (1)$$

$$D_{js}(P||N) = \int_{-\infty}^{+\infty} p(x) \log \frac{p(x)}{q(x)} dx, \text{ where } q(x) = \frac{p(x)+n(x)}{2} \quad (2)$$

The larger the divergence is the more discriminative a feature is. For Chi-square test, the null hypothesis is that the two distributions are different and thus the smaller the *p*-value, the more significant the difference is. The test results for site features and UTR features are recorded in Table 5 and Table 6. We also included the rank of features based on each divergence. Note that the rank resulted from mRMR is different from those of Kullback-Leibler divergence and Jensen-Shannon divergence. This is because that mRMR considers not only the independent discriminative power of each feature but also the correlation among features.

Table 5. statistical tests of site features

Index	Feature name	Kullback-Leibler Divergence		Jensen-Shannon Divergence		χ^2 test p-value	mRMR
		score	rank	score	rank		
1	consv_3cntxt	0.86777812	30	0.202820592	12	5.95E-97	2
2	consv_seed	1.550970461	18	0.25310581	10	7.52E-126	13
3	consv_5cntxt	0.750490992	35	0.176647445	15	1.60E-79	17
4	sm_6mer	2.01559804	12	0.519519727	1	0	1
5	sm_7mer_A1	0.834558012	33	0.261536411	9	0	4
6	sm_7mer_m1	0.879881873	29	0.2736139	8	0	6
7	sm_7mer_m8	1.147388239	24	0.341963511	7	0	5
8	sm_8mer_A1	0.508132982	43	0.165567924	19	0	7
9	sm_8mer_m1	0.475267725	46	0.155353159	23	0	9
10	to_stop_codon	2.441765729	10	0.033260162	65	0.000211042	33
11	to_ends	0.663208434	39	0.020384139	75	0.000740647	22
12	ratio_to_ends	0.020834157	103	0.005200952	99	0.252370994	44
13	nt1	0.971365378	27	0.17544652	16	5.69E-72	35
14	nt2	0.840324383	31	0.118481946	32	9.12E-23	20
15	nt3	0.77105084	34	0.103053019	34	6.60E-21	27
16	nt4	0.282048787	63	0.041126005	61	5.32E-05	43
17	nt5	0.696114016	37	0.090601346	37	8.33E-18	19
18	nt6	1.277341837	22	0.098244104	35	2.33E-22	10
19	nt7	0.373696083	56	0.068766727	47	3.88E-16	14
20	nt8	0.548499221	42	0.13008881	29	8.83E-17	23
21	nt9	0.076494781	88	0.021683228	73	2.74E-15	92
22	nt10	0.091474273	85	0.022191205	71	4.28E-08	102
23	nt11	0.207065646	70	0.051678315	53	2.69E-21	77

24	nt12	0.427710557	50	0.107291143	33	9.59E-74	21
25	nt13	0.477728391	45	0.118646099	31	6.25E-42	71
26	nt14	0.304935735	61	0.070583588	45	9.33E-25	59
27	nt15	0.120035954	80	0.027601724	68	3.27E-09	97
28	nt16	0.091046656	86	0.022181007	72	4.72E-09	61
29	nt17	0.25358407	64	0.059018925	49	1.35E-20	91
30	nt18	0.131740761	78	0.029205033	67	8.40E-09	54
31	nt19	0.108507416	83	0.025620023	69	9.39E-09	47
32	nt20	0.030357826	93	0.007296787	89	9.51E-03	84
33	2mer1	1.454458478	19	0.217399513	11	2.76E-14	45
34	2mer2	1.109423425	25	0.142954046	26	1.19E-50	41
35	2mer3	7.84083905	3	0.136393273	28	2.40E-18	51
36	2mer4	1.801004907	15	0.12910585	30	1.14E-72	30
37	2mer5	6.544491724	4	0.155560001	22	7.30E-25	34
38	2mer6	2.538557263	9	0.166387711	18	1.66E-30	25
39	2mer7	1.303954753	21	0.173144223	17	8.61E-40	36
40	2mer8	0.703671398	36	0.158377446	20	0.00E+00	48
41	2mer9	0.424976776	51	0.056524083	50	7.05E-31	72
42	2mer10	0.612456305	40	0.077197469	43	4.15E-24	83
43	2mer11	1.260992434	23	0.138084849	27	2.06E-16	50
44	2mer12	0.839620381	32	0.182570332	14	3.07E-45	62
45	2mer13	0.67743202	38	0.151409414	24	1.41E-96	37
46	2mer14	0.507471299	44	0.08656299	38	1.03E-22	74
47	2mer15	0.595257258	41	0.061570927	48	5.40E-18	76
48	2mer16	0.317953359	60	0.072076743	44	1.69E-19	82
49	2mer17	0.378491238	55	0.082767311	39	6.62E-19	73
50	2mer18	0.216033254	69	0.047186472	57	1.99E-08	79
51	2mer19	0.346674413	58	0.038662676	63	2.57E-08	65
52	rgs_match	4.885774884	7	0.454126266	2	1.5E-323	3
53	rgs_gu	0.418816115	52	0.048012096	55	1.01E-16	32
54	rgs_mismatch	1.624219113	17	0.197407996	13	2.44E-68	18
55	rgs_gap	1.785332813	16	0.080973749	41	6.95E-15	53
56	rgs_bulge	0.225154763	67	0.037525048	64	3.88E-11	63
57	rgs_bulge_nt	1.427390085	20	0.054325839	51	9.61E-09	40
58	rgs_energy	5.37287053	6	0.396751163	3	0.00E+00	12
59	rg3_match	1.831982759	14	0.151242344	25	2.18E-44	31
60	rg3_gu	0.065953486	89	0.00346257	106	4.42E-01	99
61	rg3_mismatch	3.445928207	8	0.081545494	40	2.61E-13	67
62	rg3_gap	1.094242728	26	0.041561624	60	7.73E-07	86
63	rg3_bulge	0.222955056	68	0.053008555	52	7.12E-19	57
64	rg3_bulge_nt	0.358754694	57	0.069087256	46	2.51E-17	39
65	rg3_energy	0.166010682	74	0.042690655	58	2.27E-19	24
66	rgt_match	10.69470858	1	0.362086917	6	1.18E-293	11
67	rgt_gu	0.095773914	84	0.006677135	92	1.61E-01	90
68	rgt_mismatch	5.538264744	5	0.155882428	21	7.38E-41	28
69	rgt_gap	0.91810074	28	0.079791971	42	1.78E-16	42
70	rgt_bulge	0.118690667	81	0.017991304	77	1.14E-05	88
71	rgt_bulge_nt	0.457759973	47	0.047513084	56	3.37E-07	68
72	rgt_energy	10.11446144	2	0.368076238	5	0.00E+00	16
73	acc_energy	2.085913398	11	0.369055639	4	0	8
74	cntxt_A_cntnt	0.383649528	54	0.041817218	59	9.10E-12	26
75	cntxt_C_cntnt	0.144706582	77	0.009386745	84	3.74E-02	101
76	cntxt_G_cntnt	0.304930134	62	0.039323679	62	5.19E-10	75
77	cntxt_U_cntnt	0.401228096	53	0.01746922	79	4.06E-03	80

78	cntxt_AA_cntnt	0.204602672	71	0.024945444	70	2.44E-09	60
79	cntxt_AC_cntnt	0.030308509	94	0.0086865	85	3.53E-04	38
80	cntxt_AG_cntnt	0.012581785	108	0.003054062	108	0.08910607	81
81	cntxt_AU_cntnt	0.090586599	87	0.020907011	74	7.97E-08	49
82	cntxt_CA_cntnt	0.029338332	95	0.008030575	88	4.75E-03	64
83	cntxt_CC_cntnt	0.456646035	48	0.013809972	82	0.000504596	69
84	cntxt_CG_cntnt	0.006913901	112	0.000117189	113	0.903149335	56
85	cntxt_CU_cntnt	0.021292499	102	0.003606015	105	0.064237112	70
86	cntxt_GA_cntnt	0.160024376	75	0.01014035	83	0.00330114	98
87	cntxt_GC_cntnt	0.12202816	79	0.006548877	95	0.010398961	100
88	cntxt_GG_cntnt	1.904959175	13	0.048963763	54	3.87E-14	58
89	cntxt_GU_cntnt	0.189983786	72	0.006386462	96	3.45E-02	103
90	cntxt_UA_cntnt	0.234565873	66	0.015386408	80	1.78E-05	85
91	cntxt_UC_cntnt	0.002156416	113	0.000536435	112	6.76E-01	55
92	cntxt_UG_cntnt	0.249376369	65	0.003992201	103	0.296355558	95
93	cntxt_UU_cntnt	0.341908278	59	0.030539782	66	2.20E-09	52
94	cntxt_pos_n8	0.029003846	97	0.007082567	90	2.17E-02	94
95	cntxt_pos_n7	0.013185193	107	0.00310402	107	0.328776514	106
96	cntxt_pos_n6	0.008046365	111	0.001905489	111	0.606932628	112
97	cntxt_pos_n5	0.029097631	96	0.007030172	91	0.013233053	93
98	cntxt_pos_n4	0.026659185	100	0.006326836	97	0.032169227	109
99	cntxt_pos_n3	0.012106263	109	0.002977402	109	0.212191137	111
100	cntxt_pos_n2	0.018317939	104	0.004439047	101	0.076481015	104
101	cntxt_pos_n1	0.167964236	73	0.004938317	100	0.158032852	108
102	cntxt_pos_n0	0.157054497	76	0.020335344	76	5.18E-07	66
103	cntxt_pos_p1	0.1136777	82	0.017608503	78	3.32E-07	29
104	cntxt_pos_r1	0.432186485	49	0.096576671	36	6.82E-35	15
105	cntxt_pos_r2	0.035000936	91	0.00847264	86	3.47E-03	78
106	cntxt_pos_r3	0.015236682	106	0.003821404	104	0.081360535	113
107	cntxt_pos_r4	0.060329539	90	0.014760487	81	1.76E-05	46
108	cntxt_pos_r5	0.016479689	105	0.004021703	102	8.89E-02	96
109	cntxt_pos_r6	0.026786286	99	0.006646065	94	0.010093675	107
110	cntxt_pos_r7	0.027750883	98	0.006667523	93	0.015172783	110
111	cntxt_pos_r8	0.008245467	110	0.002060143	110	0.313469199	89
112	cntxt_pos_r9	0.032501088	92	0.008095066	87	0.002809335	87
113	cntxt_pos_r10	0.025282908	101	0.006220051	98	0.015739625	105

Table 6. statistical tests of UTR features

Index	Feature name	Kullback-Leibler Divergence		Jensen-Shannon Divergence		χ^2 test	mRMR
		score	rank	score	rank	p-value	
1	utr_len	0.296138525	9	0.090666411	4	9.88E-15	18
2	psite_dens	0.386280146	6	0.03084399	21	1.87E-16	23
3	max_partial_psite_num	0.149384251	22	0.014817448	26	7.17E-11	24
4	pos_site_dens	0.363401103	7	0.040485379	15	1.13E-86	5
5	max_partial_pos_site_num	0.349544201	8	0.063802623	8	1.23E-63	4
6	total_pos_score	0.470628209	4	0.147695304	1	1.35E-198	2
7	psite_num	0.198889206	15	0.034014427	18	6.79E-22	19
8	pos_site_num	0.391843812	5	0.041553768	12	1.86E-110	3
9	top_score	0.698130776	2	0.04108018	13	2.70E-231	1
10	psite_num_6mer	0.158568098	20	0.005803861	29	1.98E-03	28
11	pos_site_num_6mer	0.158568098	21	0.002129584	30	1.98E-03	29
12	top_score_6mer	2.2758712	1	0.025174242	24	3.89E-01	14

13	psite_num_7mer_A1	0.118171366	23	0.064337846	6	7.01E-46	16
14	pos_site_num_7mer_A1	0.118171366	24	0.03465331	16	7.01E-46	17
15	top_score_7mer_A1	0.255939795	11	0.050310791	11	7.01E-46	13
16	psite_num_7mer_m1	0.10775924	25	0.03465331	17	1.44E-40	20
17	pos_site_num_7mer_m1	0.10775924	26	0.031226941	19	1.44E-40	21
18	top_score_7mer_m1	0.2636244	10	0.040744283	14	1.44E-40	12
19	psite_num_7mer_m8	0.069606233	28	0.016449273	25	2.28E-17	26
20	pos_site_num_7mer_m8	0.069606233	29	0.014817448	27	2.28E-17	27
21	top_score_7mer_m8	0.091320624	27	0.03084399	20	2.28E-17	25
22	psite_num_8mer_A1	0.205942877	12	0.130415629	2	6.00E-162	6
23	pos_site_num_8mer_A1	0.205942877	13	0.100148516	3	6.00E-162	7
24	top_score_8mer_A1	0.204839793	14	0.064337846	7	6.00E-162	8
25	psite_num_8mer_m1	0.165061748	17	0.08682064	5	4.18E-108	9
26	pos_site_num_8mer_m1	0.165061748	18	0.050799375	9	4.18E-108	10
27	top_score_8mer_m1	0.164006127	19	0.050799375	10	4.18E-108	11
28	psite_num_other	0.183431771	16	0.027350187	22	1.54E-18	22
29	pos_site_num_other	0.007023711	30	0.005803861	28	1.15E-08	30
30	top_score_other	0.575297226	3	0.027316603	23	8.35E-15	15

S.10. PERFORMANCE OF SVMICRO VS. SITE-UTR MODEL

The performance improvement due to UTR-SVM is investigated. To this end, a site-SVM based predictor is constructed, which follows the structure of TargetScan and assigns a score *S* to a UTR that equals to the sum of all positive site-SVM scores for this UTR. If no site is predicted positive for a UTR, the score is set 0. The ROC curve of the site-SVM predictor based on the UTR training data is plotted in Fig 14 together with that of the SVMicrO. As expected, site information plays the most important role in target prediction as the performance of site predictor accounts for the major portion of the overall performance of SVMicrO. However, UTR SVM does improve both the sensitivity and specificity of the site-SVM based predictor, indicating the necessity to consider UTR features in target prediction.

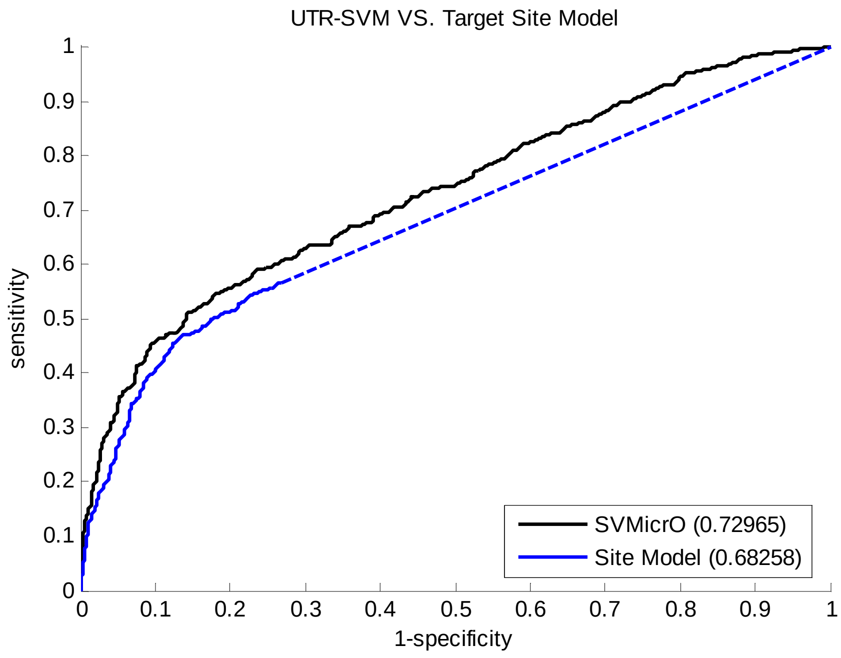

Fig. 14. ROC curves of SVMicrO and the site-SVM based predictor.

S.11. EVALUATION BASED ON THE PROTEOMICS DATA

To demonstrate the robustness of prediction, we carried out the prediction of 5 more miRs (miR-155, hsa-let-7b, hsa-miR-16, and hsa-miR-30a), for which the proteomic data are available in (Selbach, et al., 2008). The cumulative fold changes of different number of top ranked predictions for each miRNA are summarized in Figs. 12-15, respectively. In all cases, SVMicrO achieves the largest down-fold for three of the 4 miRs by top 300, indicating a better sensitivity. For the performance of the top 200 predictions, SVMicrO has achieved consistently among the highest cumulative down-fold; this suggests the better precision of the algorithm.

In addition, the significance of the cumulative fold changes at different top rankings for the tested algorithms is assessed by random permutation (Table 7-12). For example, to calculate the *p*-values of the cumulative sums for the top 25 ranked predictions of miR-1 targets by an algorithm, the prediction ranking is randomly permutated 10,000,000 times, and for each permutation the cumulative sum of the top 25 protein fold changes is recorded. The *p*-value is then calculated as the percentage of the permutated sums that are less than the cumulative sum of the top predictions by that algorithm.

Table 7. **C**umulative sum of protein fold change and p-value for different number of top ranked predictions of hsa-miR-1.

Top number	SVMicro		TargetScan		miRanda		MirTarget		PicTar		PITA	
	CF	p-value	CF	p-value	CF	p-value	CF	p-value	CF	p-value	CF	p-value
25	-5.211	0.001389	-6.765	6.58E-05	-2.134	0.145628	-6.21	0.000208	-4.879	0.002509	-2.014	0.165675
50	-11.305	1.90E-06	-9.475	5.96E-05	-6.327	0.006981	-12.68	< 1E-07	-12.095	5.00E-07	-6.788	0.003827
100	-16.045	2.30E-06	-11.648	0.000776	-12.08	0.000469	-13.261	0.00011	-16.321	1.90E-06	-9.352	0.008214
200	-23.416	2.70E-06	-16.562	0.001498	-20.605	3.93E-05	-13.261	0.013928	-16.321	0.001806	-14.652	0.005932
300	-33.257	< 1E-07	-16.562	0.014624	-20.605	0.001833	-13.261	0.049026	-16.321	0.016202	-18.805	0.005019

Table 8. **C**umulative sum of protein fold change and p-value for different number of top ranked predictions of hsa-miR-124.

Top number	SVMicro		TargetScan		miRanda		MirTarget		PicTar		PITA	
	CF	p-value	CF	p-value	CF	p-value	CF	p-value	CF	p-value	CF	p-value
25	-16.901	< 1E-07	-13.422	6.10E-06	-3.949	0.1138358	-11.596	6.54E-05	-12.829	1.29E-05	-5.977	0.024792
50	-30.586	< 1E-07	-24.905	< 1E-07	-12.547	1.76E-03	-15.022	0.0001918	-12.787	1.43E-03	-18.045	9.40E-06
100	-35.582	< 1E-07	-40.527	< 1E-07	-24.729	1.50E-05	-15.022	0.0113462	-15.165	1.06E-02	-26.945	2.60E-06
200	-41.643	3.00E-07	-42.46	2.00E-07	-24.729	4.64E-03	-15.022	0.0986528	-15.165	0.095504	-43.823	1.00E-07
300	-56.157	< 1E-07	-42.46	3.80E-05	-24.729	0.0290952	-15.022	0.1935084	-15.165	0.189559	-47.241	1.60E-06

Table 9. **C**umulative sum of protein fold change and p-value for different number of top ranked predictions of hsa-let-7b.

Top number	SVMicro		TargetScan		miRanda		MirTarget		PicTar		PITA	
	CF	p-value	CF	p-value	CF	p-value	CF	p-value	CF	p-value	CF	p-value
25	-6.07	< 1E-07	-0.8	3.44E-01	-3.5	9.68E-04	-6.55	< 1E-07	-4.87	6.50E-06	-1.11	0.2364556
50	-9.55	< 1E-07	-0.8	5.01E-01	-5.8	1.73E-04	-6.55	2.27E-05	-10.33	< 1E-07	-2.63	9.35E-02
100	-16.24	< 1E-07	-0.8	6.44E-01	-10.94	7.00E-07	-6.55	0.0054987	-11.75	< 1E-07	-5.66	1.85E-02
200	-21.11	< 1E-07	-0.8	7.73E-01	-14.78	7.80E-06	-6.55	0.1065933	-11.75	0.0007699	-13.76	4.23E-05
300	-35.87	< 1E-07	-0.8	8.45E-01	-14.78	0.0010575	-6.55	2.79E-01	-11.75	1.60E-02	-15.64	4.16E-04

Table 10. **C**umulative sum of protein fold change and p-value for different number of top ranked predictions of hsa-miR-16.

Top number	SVMicro		TargetScan		miRanda		MirTarget		PicTar		PITA	
	CF	p-value	CF	p-value	CF	p-value	CF	p-value	CF	p-value	CF	p-value
25	-10.97	< 1E-07	-8.29	4.30E-06	-6.4	0.0001587	-9.55	3.00E-07	-8.53	3.00E-06	2.11	0.8474938
50	-19.74	< 1E-07	-12.3	6.00E-07	-11.68	1.40E-06	-15.88	< 1E-07	-13.48	< 1E-07	-3.1	0.0472842
100	-32.8	< 1E-07	-13.66	3.70E-06	-21.93	< 1E-07	-31.02	< 1E-07	-30.79	< 1E-07	-6.43	0.0075444
200	-54.29	< 1E-07	-13.38	0.0001517	-36.12	< 1E-07	-31.02	< 1E-07	-31.4	< 1E-07	-12.06	0.0004281

300	-65.21	< 1E-07	-13.38	0.0004271	-36.55	< 1E-07	-31.02	< 1E-07	-31.4	< 1E-07	-17.15	2.94E-05

Table 11. Cumulative sum of protein fold change and p-value for different number of top ranked predictions of hsa-miR-30a.

Top number	SVMicro CF	p-value	TargetScan CF	p-value	miRanda CF	p-value	MirTarget CF	p-value	PicTar CF	p-value	PITA CF	p-value
25	-9.34	< 1E-07	-0.15	4.69E-01	-7.12	1.00E-07	-7.36	1.00E-07	-8.63	< 1E-07	-0.02	0.5168371
50	-15.05	< 1E-07	-1.78	1.50E-01	-10.66	< 1E-07	-12.91	< 1E-07	-13.39	< 1E-07	-2.12	1.08E-01
100	-25.57	< 1E-07	-1.91	2.13E-01	-14.54	< 1E-07	-21.36	< 1E-07	-18.84	< 1E-07	-5.15	1.54E-02
200	-31.74	< 1E-07	-1.91	2.56E-01	-24.01	< 1E-07	-23.04	< 1E-07	-19.58	< 1E-07	-6.09	3.32E-02
300	-34.42	< 1E-07	-1.91	2.60E-01	-24.45	< 1E-07	-23.04	< 1E-07	-19.58	6.00E-07	-5.6	7.24E-02

Table 12. Cumulative sum of protein fold change and p-value for different number of top ranked predictions of hsa-miR-155.

Top number	SVMicro CF	p-value	TargetScan CF	p-value	miRanda CF	p-value	MirTarget CF	p-value	PicTar CF	p-value	PITA CF	p-value
25	-13.17	< 1E-07	-20.43	< 1E-07	-11.49	< 1E-07	-17.63	< 1E-07	-10.7	2.00E-07	-3.31	0.0456311
50	-20.44	< 1E-07	-31.76	< 1E-07	-25.14	< 1E-07	-24.06	< 1E-07	-13.99	2.00E-07	-8.09	1.60E-03
100	-44.34	< 1E-07	-49.34	< 1E-07	-32.04	< 1E-07	-31.25	< 1E-07	-13.99	1.87E-04	-19.57	1.00E-07
200	-71	< 1E-07	-87.21	< 1E-07	-40.87	< 1E-07	-31.25	< 1E-07	-13.99	0.0203551	-33.34	< 1E-07
300	-103.81	< 1E-07	-97.15	< 1E-07	-40.87	< 1E-07	-31.25	3.50E-06	-13.99	0.1049568	-42.94	< 1E-07

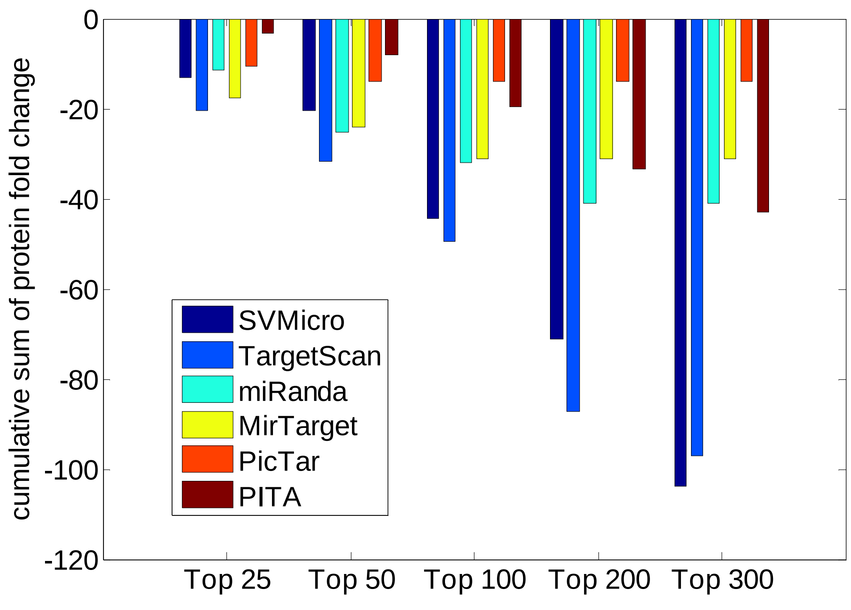

Fig. 15. Cumulative sum of protein fold change for different number of top ranked predictions of miR-155.

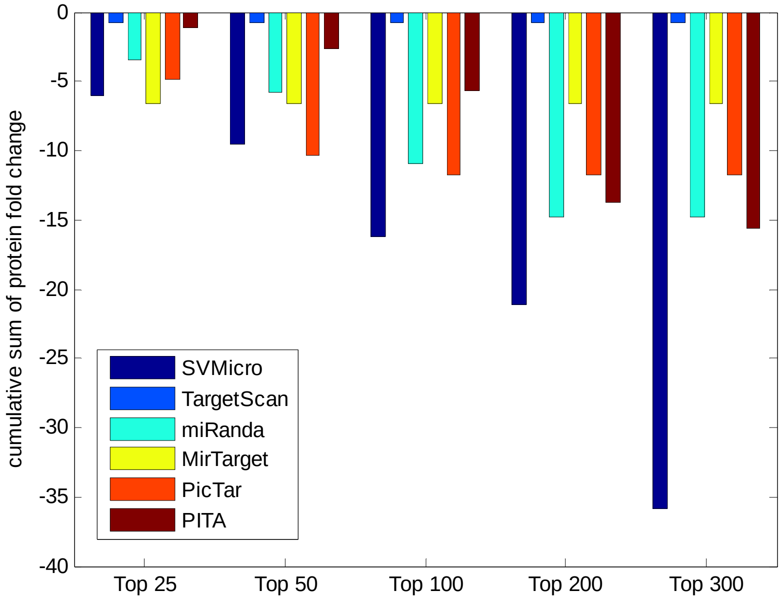

Fig. 16. Cumulative sum of protein fold change for different number of top ranked predictions of miR-let-7b.

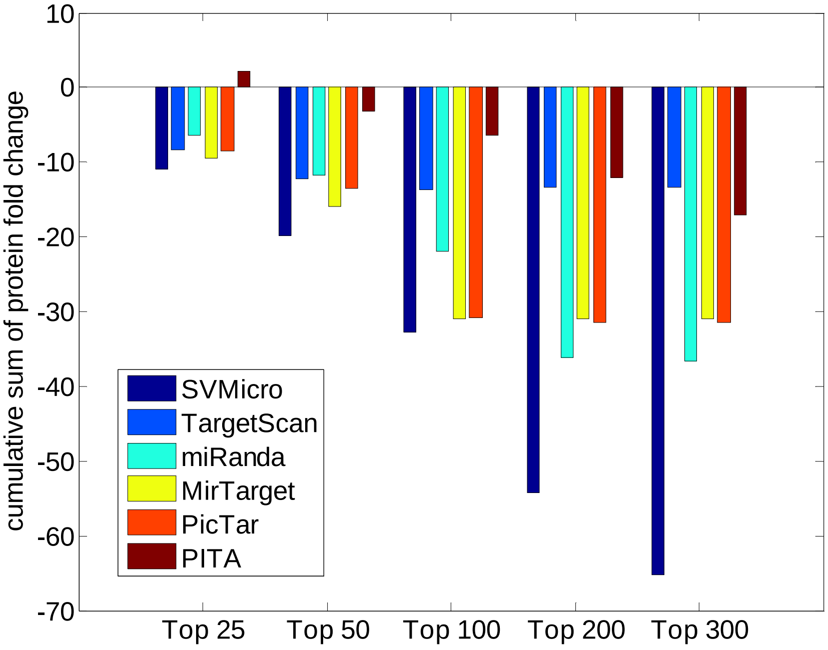

Fig. 17. Cumulative sum of protein fold change for different number of top ranked predictions of miR-16.

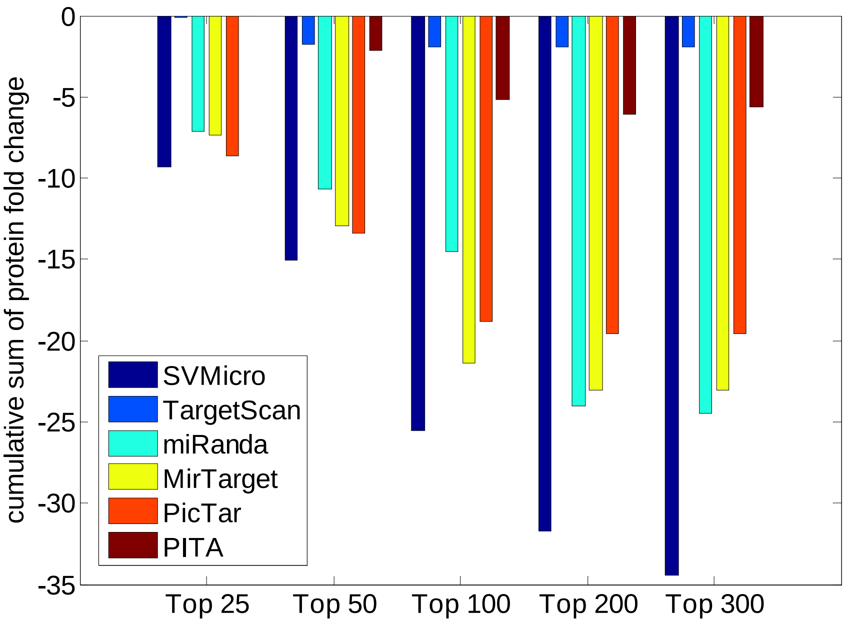

Fig. 18. Cumulative sum of protein fold change for different number of top ranked predictions of miR-30a.
